# Feature Signature Discovery for Autism Detection: An Automated Machine Learning Based Feature Ranking Framework

**DOI:** 10.1155/2023/6330002

**Published:** 2023-01-04

**Authors:** Shomona Gracia Jacob, Majdi Mohammed Bait Ali Sulaiman, Bensujin Bennet

**Affiliations:** ^1^University of Technology and Applied Sciences, Nizwa, Postal Code: 611, Oman; ^2^University of Technology and Applied Sciences, Salalah, Postal Code: 211, Oman

## Abstract

Autism spectrum disorder is the most used umbrella term for a myriad of neuro-degenerative/developmental conditions typified by inappropriate social behavior, lack of communication/comprehension skills, and restricted mental and emotional maturity. The intriguing factor of this disorder is attributed to the fact that it can be detected only by close monitoring of developmental milestones after childbirth. Moreover, the exact causes for the occurrence of this neurodevelopmental condition are still unknown. Besides, autism is prevalent across individuals irrespective of ethnicity, genetic/familial history, and economic/educational background. Although research suggests that autism is genetic in nature and early detection of this disorder can greatly enhance the independent lifestyle and societal adaptability of affected individuals, there is still a great dearth of information to support the statement of proven facts and figures. This research work places emphasis on the application of automated machine learning incorporated with feature ranking techniques to generate significant feature signatures for the early detection of autism. Publicly available datasets based on the Q-chat scores of individuals across diverse age groups—toddlers, children, adolescents, and adults have been employed in this study. A machine learning framework based on automated hyperparameter optimization is proposed in this work to rank the potential nonclinical markers for autism. Moreover, this study aimed at ranking the AutoML models based on Mathew's correlation coefficient and balanced accuracy via which nonclinical markers were identified from these datasets. Besides, the feature signatures and their significance in distinguishing between classes are being reported for the first time in autism detection. The proposed framework yielded ∼90% MCC and ∼95% balanced accuracy across all four age groups of autism datasets. Deep learning approaches have yielded a maximum of 92.7% accuracy on the same datasets but are limited in their ability to extract significant markers, have not reported on MCC for unbalanced data, and cannot adapt automatically to new data entries. However, AutoML approaches are more flexible, easier to implement, and provide automated optimization, thereby yielding the highest accuracy with minimal user intervention.

## 1. Introduction

Developmental disorders are chronic disabilities and in recent years autism has gained importance across the globe owing to the increasing count of families facing a dilemma while raising affected children [1]. This has caused anxiety about the future of autistic children, their acceptance in society, personal and professional competence, and the need for individual attention. Research in the sphere of autism disorders suggests that autism, when diagnosed early, can be treated effectively, although a complete cure at present is considered impossible. A research team at the University of Missouri recorded the results of their research that stated that autistic children and healthy controls share quite a few typical facial attributes. This study was based on the images distributed by the Kaggle database [2, 3].

Detecting autism sans delay is pivotal to choosing the most appropriate course of therapy, deciding on the level of attention and personal care for the child, managing patient expenses, and prioritizing the nature of schooling and education. The societal stigma associated with Autism Spectrum Disorders (ASD) has escalated the mental trauma faced by families. Early detection of this neurological condition is expected to assist in timely therapy and nurturing for the affected child and family [4]. Complete dependence on physicians and scientists to research and analyze the causes, symptoms, nature, and therapeutic measures to treat ASD is a labor-intensive task that would consume substantial resources in terms of money, time, and expertise [5, 6]. This paper proposes a hybrid computational framework based on automated machine learning techniques to unveil the most crucial factors that can enable early and timely detection of the disorder.

Artificial intelligence (AI) solutions are influencing every sphere of life ranging from finance and education to the field of medicine and defense. Machine learning, one of the major building blocks of AI, has been studied and diverse techniques have been proposed in the recent past for computer-aided diagnosis of autism [4–8]. Interactive mobile and web applications have been deployed as computerized personal assistants to support the convalescence and treatment of autistic patients [9–11]. Several scientists have proposed machine learning algorithms for the classification of neuro-degenerative ailments, such as schizophrenia [12–14], dementia, depression, and other psychiatric disorders [15–17] from MRI data. Most of the previous work is related to classifying autism from the brain or facial images of individuals.

Machine learning techniques have been proposed in the past for autism classification from clinical datasets obtained by collating real-time questionnaires from parents and healthcare workers. These have been made publicly available as four different nonclinical ASD datasets. Recent research has now delved into the use of deep learning techniques for image classification and text analysis. Many recent reports in the literature, have compared the performance of conventional and deep learning approaches for autism classification [12–18]. Presently, state-of-the-art methods have placed emphasis on auto-machine learning [19, 20], a new domain of machine learning that automates the process of hyperparameter selection, feature ranking, and evaluation protocols that aim at obtaining the highest accuracy in distinguishing between healthy and affected patients while also disclosing the most important feature combinations that contribute to the accurate categorization of affected individuals.

Autism needs to be initially identified by the caretakers of the infant/child. Hence, nonclinical marker detection plays a crucial role in enabling parents/family members to easily identify the level of developmental delay in the child [1–3]. The development of a machine learning-based computational framework that would reveal the potential nonclinical markers for autism would enable even a medical inexpert to identify the possibility of autism in their ward and seek early medical advice. This research work focuses on achieving the following main objectives: (i) propose an AutoML-based computational framework that combines the best feature ranking and classification approach to generate high classification accuracy. (ii) Identify the role of potential nonclinical markers in the order of increasing importance (feature signatures) that can detect autism with minimal, yet significant information. (iii) Compare the use of traditional, deep learning, and AutoML techniques in classifying autism from nonclinical data.

The organization of the research article is planned as follows: this section is followed by the state-of-the-art on the recent research findings in the sphere of computational classification of ASD. Later, the article describes the materials and proposed methods carried out in this work, which is followed by a detailed description of the experimental results and analysis. Discussions on the findings of this research are presented after the results followed by the conclusions from the work.

## 2. Literature Survey

Autism Spectrum Disorders have been studied by researchers for ages and the findings indicate that ASD diagnosis adopted two main approaches—categorical and dimensional. Volkmar et al. [21] stated that for clinical practice in real-time settings, the diagnosis of autism was inclined towards adopting an ideographic approach, that placed emphasis on the characteristics and symptoms of the specific individual. The authors observed that in recent years, categorical approaches had resorted to the use of Research Diagnostic Criteria (RDC) and had great value for maintaining clinical records for statistical analysis. However, the downsides included handling some critical challenges like defining thresholds for certain conditions, linking symptoms with comorbidities, recognizing developmental changes, etc. Hence, this motivated the authors to focus on detecting the potential features from data obtained by observation and questionnaires that could enable caretakers of children to identify autism early.

Ahmed et al. [22] proposed a classification prototype that was a fusion of a restricted Boltzmann machine (RBM) and support vector machine (SVM), the former being used for feature selection from fMRI images while the latter was utilized for binary classification between healthy and affected individuals. A myriad of data processing steps that included slice time correction and normalization were also performed before generating the machine learning models. Their dataset comprised of 105 typical control (TC) and 79 ASD subjects from the authentic ABIDE data repository. Their findings suggested that the amalgamation of RBM and SVM methods may be applied as a future tool to diagnose ASD. The author's report does not suggest a comparison with other traditional/deep learning models, and neither does the work report on the ranking of facial features and how they are distinguished between the target classes.

Mohanty et al. [23] reported their classification results on publicly accessible and authenticated ASD datasets from the UCI machine learning (ML) repository and Kaggle. The authors proposed a deep learning-based classifier on the child data set gathered from the UCI repository. They analyzed two types of data—complete data and data with missing values. The diffusion mapping feature selection method was utilized, and classification was done by implementing a deep neural network classifier. Their proposed method yielded an accuracy of 94% on complete data and 92% on missing data, which was higher than the traditional machine learning models. However, their work did not report on the importance of specific markers for autism detection but rather focussed only on the classification performance. Moreover, deep learning approaches require machine learning expertise for the accurate implementation and proper hyperparameter optimization to generate higher accuracy.

Alsaade et al. [1] proposed a novel deep learning-based system, designed to detect autism from facial images. The authors identified the need for a unique technology that could extract significant facial features/patterns to distinguish between autistic and nonautistic facial images. They proposed a simple web application using a deep learning approach based on a convolutional neural network with transfer learning and the flask framework. Xception, Visual Geometry Group Network-19 (VGG19), and NASNetMobile were chosen to be the models that were pretrained and utilized for categorization. The publicly available facial image dataset from Kaggle was utilized for this purpose. Their results recorded that Xception was the most accurate with 91% correctly classified samples, followed by VGG19 (80%) and NASNetMobile (78%). This work is limited by the fact that the facial images available in the Kaggle repository belong to children aged 5 and above. Moreover, this system will not be interpretable to amateur child caretakers who will remain unaware of their ward's neuro-developmental state.

Several studies were undertaken on classifying ASD from images through traditional machine learning models, hybrid models that infuse feature selection with classification, deep learning models, and AutoML models [20, 23–27]. A concise review of the recent research on ASD classification is depicted in Table 1.

However, thus far in research, there have been only comparisons of different ML models based on their performance in classifying autism using the UCI ML and Kaggle toddler datasets [28–31]. Both deep learning and traditional methods have been applied and their results have generated reasonable accuracy. However, the methods portrayed latent inadequacy since (i) the models required human intelligence and utilized trial-error experiments for hyperparameter optimization, (ii) research on AutoML models for classifying autism across all age groups has not been reported thus far, and (iii) most of the previous work in the literature have relied on heavy data preprocessing which is perceived to be the reason that their models could not yield high Mathew's correlation coefficient on unbalanced datasets such as the toddler dataset from Kaggle.

This research work contributes to the current state of autism classification through machine learning models by aiming at the following three main objectives: (i) Design of a hybrid AutoML-based machine learning framework that interprets the role of potential attributes (feature signatures) for detecting autism. (ii) Automation of algorithmic parameters during execution such that in the event of new data being added to the training set, the use of AutoML approaches would optimize the model parameters and tune the functions to yield the highest performance. (iii) Compare the performance of traditional, deep, and AutoML approaches in autism classification on all 4 datasets employed in this study by utilizing metrics suitable for both balanced and unbalanced datasets.

## 3. Materials and Methods

### 3.1. Materials

This study concentrates on four autism datasets (i) the child autism dataset—UCI, (ii) the adolescent autism dataset—UCI, (iii) the adult dataset—UCI, and (iv) the toddler dataset—Kaggle. Thabtah [29, 30] provided the data to screen autism across all age groups.  Table 2 summarizes the description of the attributes of toddlers.

The authors noted that most of the predictor attributes were similar in all four datasets. The type of data is diverse across the datasets. All datasets have missing values. The UCI dataset incorporates one additional feature of including individuals who have already used the screening app. One of the objectives of this research is to find the most crucial questions/observations that could lead to early and accurate detection of autism in a noninvasive and less stigmatic manner.

**Figure 1:**
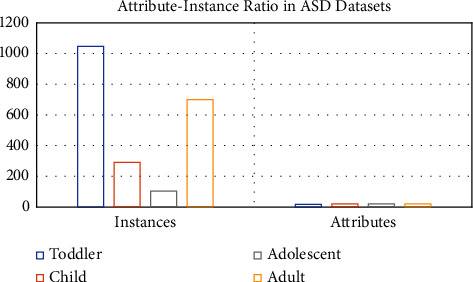
Data distribution across the autism datasets.

The number of instances and attributes for all the datasets included in this study is graphically represented in Figure 1.

The software platforms and machine learning models based on automated machine learning are described in the following subsection.

### 3.2. Methods

The software suite utilized in this research is JadBio Automated Machine Learning Platform [32]. AutoML tools have been utilized in recent years to generate robust predictive and diagnostic models that do not carry the traits of the black box approach. The most recently unveiled Just Add Data Bio (JADBio) platform (https://www.jadbio.com) is an AutoML technology that is very flexible and user-friendly with a highly interpretable interface that generates exhaustive reports on every machine learning task [33]. They are readily applicable to data of diverse natures and have inbuilt automated data preprocessing techniques that lead to the generation of highly accurate predictive models and feature signatures.

To the best of our knowledge, no other AutoML platform can identify small-size feature signatures or generate such accurate interpretable results in less time.

**Figure 2:**
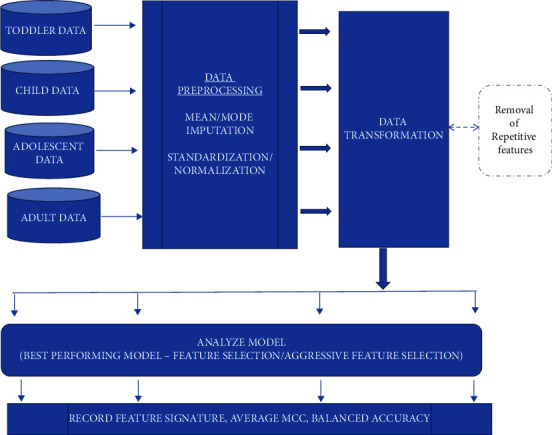
Proposed AutoML framework for feature signature discovery—training phase.

**Figure 3:**
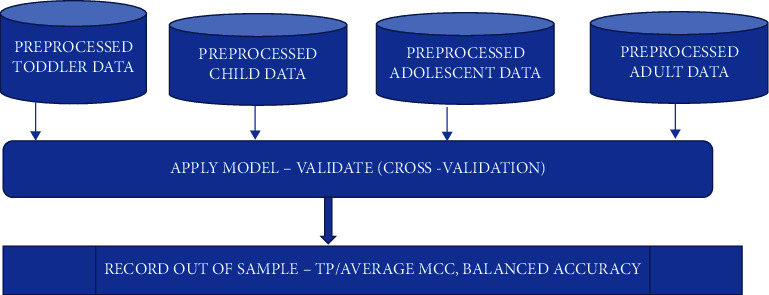
Proposed AutoML framework for feature signature discovery—validation phase.

The AutoML framework proposed in this article is portrayed in Figures 2 and 3. The framework comprised of 2 phases, training phase and validation phase. Initially the data was uploaded following which the suite provided an option to transform data if necessary. In all datasets, sample ID and sum of Q-chat score results were removed as part of the transformation. This was done to identify which questions in the questionnaire held most significance in the order of increasing importance according to the best performing AutoML model.

The transformed data were then loaded onto the AutoML software suite, and the analysis commenced after the software acquired certain user inputs such as the option to choose between interpretable models or the best-performing models. The users can also choose to perform feature selection or aggressive feature selection. The choice of evaluation metric to rate the performance of the models, and the number of CPU cores for large data was also available. In this work, the authors chose to analyze using the best-performing model with feature selection and aggressive feature selection. Aggressive feature selection differs from feature selection as it places focus on generating the most crucial and small-sized feature signature even at the loss of predictor performance.

The proposed framework commenced with the data preprocessing phase, and as is evident from Table 1, the data comprised both numerical and categorical values. Hence, to replace the missing values, the mean of the available numerical values and the mode (most frequently occurring) of the available categorical values were substituted [34]. This was followed by standardization and normalization to ensure that the features of the input data set were not constrained to a particular range and that large values in the data set did not impact the training process adversely [35].

The analysis of the model began with feature selection. AutoML in JADBio implemented the SES (statistically equivalent signature) and LASSO (least absolute shrinkage and selection operator) methods. The SES algorithm works on the principle of Bayesian networks' constraint-based learning [36].

The SES method manages to unearth multiple predictive feature subsets whose performances are statistically equivalent [36].

LASSO is a linear model that uses the following cost function:

(1) $$\begin{array}{c} Cost=\frac{1}{{2N}_{training}}+\overset{N_{training}}{\underset{i=1}{\sum }}{\left(y_{real}^{i}-y_{pred}^{i}\right)}^{2}+\alpha \overset{n}{\underset{j=1}{\sum }}\left\lceil a_{j}\right\rceil \text{,} \end{array}$$

where *a*_*j*_ is the coefficient of the *j*^th^ feature. The final term is called the *L*_1_ penalty and *α* is a hyperparameter that tunes the intensity of this penalty term. The higher the coefficient of a feature, the higher the value of the cost function.

LASSO feature selection aims to optimize the cost function by minimizing the absolute values of the coefficients [37]. This feature selection is applicable to data that were scaled using standardization for optimum results. Since this machine learning technique follows an automated approach, the *α* hyperparameter value is automatically selected by a cross-validation approach. Hence, when the coefficient of any feature is 0, it is discarded. The model fits a lasso regression on a scaled version of the data and retains only those features whose coefficient is different from 0.

Feature selection is followed by classification and the best performing model was SVM of type C-SVC using a linear/polynomial/radial basis function kernel with cost and gamma hyperparameters set to 1 [38]. The detailed results are discussed in the next section. SVM has been very widely used in the medical literature to solve diverse classification problems. An exhaustive description of the algorithmic principles and hyperparameters is available in this article [39–41].

## 4. Experimental Results and Performance Analysis

The results of this work are reviewed in three sections. The first section elaborates on the performance metrics and the evaluation methods that were adopted in this study. The second section depicts the feature selection and classification results based on the selected metrics. The third section describes the comparative performance of AutoML classifiers with previously reported records on the same autism datasets.

### 4.1. Evaluation Metrics and Methods

**Figure 4:**
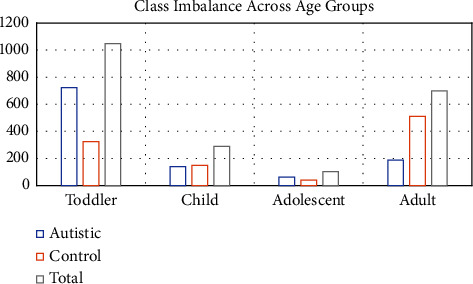
Class distribution across the ASD datasets.

The standard metrics for unbalanced data were adopted to rate the AutoML models in this study. It can be noted from the graph in Figure 4 that the toddler and adult datasets have a high-class imbalance between autistic and control subjects. Hence, the authors proposed to use MCC [42] and balanced accuracy which has been reported in the literature to be the most accurate in predicting datasets with class imbalance and those with balanced data as well. This is the first time in the literature that these metrics have been adopted to classify ASD datasets.

Both these metrics are computed using the confusion matrix that calculates the number of true positives (TP), false positives (FP), true negatives (TN), and false negatives (FN). In this study, if the subject is nonautistic, it is considered a positive case. Based on these terms, the MCC and balanced accuracy were calculated as follows [43, 44]:

(2) $$\begin{array}{c} \propto_{MCC}=\frac{\left(TP*TN\right)-\left(FP*FN\right)}{\sqrt{\left(TP+FN\right)*\left(TN+FP\right)*\left(TP+FP\right)*\left(TN+FN\right)}}\text{,}\\ \propto_{Bacc}=\frac{TP}{TP+FN}+\frac{TN}{TN+FP}\text{.} \end{array}$$

The performance evaluation method adopted in this work was, a repeated 10-fold cross-validation technique, wherein the training dataset was divided into 10 groups (folds) and during each epoch, 9 folds were used for training the model, and one fold was used to test the trained model. This was repeated 10 times and the average MCC and balanced accuracy have been reported. This is a standard statistical method used in data mining and machine learning for evaluating classifier performance. The standard confidence interval was set to 95% while performing the experiments.

### 4.2. Feature Signature and Classification

Feature signature is the combination of features from the original dataset that are highly predictive of the target class. The cumulative results of the proposed AutoML framework are tabulated in Table 3.

The feature signature is generated by AutoML predictors in the order of increasing importance. It is to be recorded based on the tabulated performance metrics in Table 3 that the feature signature generated by the feature selection method on all four datasets has yielded high MCC and balanced accuracy, almost comparable to the performance of the full feature selector. However, in the case of the adolescent dataset, the MCC shows considerable variation, especially while implementing aggressive feature selection.

**Figure 5:**
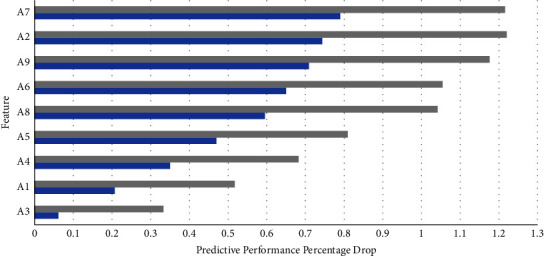
Feature signature and model predictive performance on toddler dataset.

**Figure 6:**
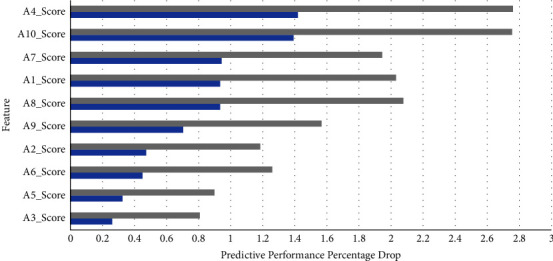
Feature signature and model predictive performance on child dataset.

To ascertain the contribution of each individual component to the feature signature, the authors attempted to identify the role of each feature in enhancing predictive performance. Since the aggressive feature selection methods try to minimize the feature signature size at the cost of the model's predictive performance, as witnessed from the results in Table 1, the authors focussed their attention on the best-performing AutoML models with feature selection alone. Figures 5 and 6 portray the reduction in loss of predictive performance as each attribute is added to the feature signature in the decreasing order of importance on the toddler and child datasets, respectively.

The feature signature on the toddler and child dataset denotes that there is very little space for improving the performance as both graphs indicate a maximum reduction of ∼0.2% on the predictor performance.

**Figure 7:**
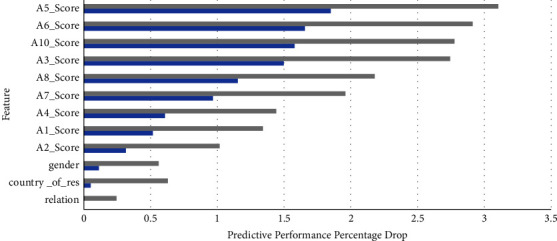
Feature signature and model predictive performance on the adolescent dataset.

**Figure 8:**
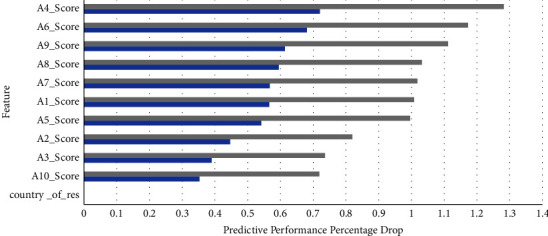
Feature signature and model predictive performance on the adult dataset.

The country of residence is a new attribute added to the feature signature in the age groups over 11 as noted from Figures 7 and 8, that is contributing to almost maximum MCC, while almost all Q-chat attributes seem to be playing a very prominent role in both these feature signatures.

It is evident from the feature signatures generated across all age groups that the questions in the Q-chat alone will suffice to detect autism in young children based on observation of behavior with more than 90% accuracy. Moreover, the use of the autism screening apps, ethnicity, or the existence of autism in the family has not surfaced as a ranked feature in any of the four feature signatures that generated high predictive performance.

Recent work in the literature that has reported on the use of the JadBio AutoML framework on other biological datasets has recorded only the training estimates. The authors in this work proceed to validate the performance metrics generated by JADBio using validation data. Four metrics to test the validity of the generated AutoML models across all four datasets have been utilized as seen in Table 4.

The OUT OF SAMPLE TESTING (OOST) metrics have been recorded for the first time on Autism data. This is a measure of how the model predicts a sample that it has not been trained on, that is, totally new test data. The lesser false predictions (FP and FN) in OOST, the more robust the model.

The tabulated results clearly reveal that the OOS metrics have very few misclassifications across all age groups. Even aggressive feature selection models have depicted a classification performance comparable to feature selection-based models on the validation data.

### 4.3. Comparison to Previous Work

Since no previous literature is available on AutoML models for autism classification from nonclinical data and most of the existing literature has reported only on the accuracy using traditional and deep learning models, the authors make a comparative performance based on the accuracy of the AutoML models on the ASD datasets.

The comparative study of results in Table 5 clearly reveals that the AutoML methods are most suitable for improving predictor performance on balanced and unbalanced data. The accuracy of the proposed methods was obtained by using repeated 10-fold cross-validation. However, since earlier results have reported a higher accuracy of ∼100% on the child dataset, the authors attempted to validate the AutoML model against a test dataset. The prediction accuracy was close to 100% using the AutoML model on the child dataset when all the features were included for classification.

## 5. Discussion

Autism-affected individuals irrespective of their age, gender, social status, and educational backgrounds, are subjected to intense social stigma and this affects not only the individual but also their families and the society at large. This research work was undertaken to identify the exact combination of features that could enable early detection of autism, based on questionnaires to caretakers of infants/toddlers who are at high-risk. Research has suggested possible links towards certain high-risk factors like premature gestation delivery, adopting improper delivery mechanisms, unprescribed medical dosages during pregnancy, increased maternal age, and genetic factors [45, 46].

The feature signatures generated for autism detection across all age groups clearly reveal that questions on social interaction and general behavior would suffice to identify the possibility of autism development. This would surely enable caretakers and physicians to ensure that every child is monitored during regular clinic visits and general health checks for behavioral and social interaction abilities.

This discussion is restricted to identifying the feature signatures for toddler and child datasets since adolescents and adults, by general behavior, reveal their neurodegenerative status. However, those datasets were included in this study for validating the AutoML classifier performance. It is also to be noted that although multiple signatures could be generated for a single dataset, the proposed AutoML models generated only a single distinct signature for each of the four datasets.

  The feature signature for toddlers: A9, A7, A2, A6, A5, A4, A8, A1, and A3
  The feature signature for children: A10, A4, A9, A5, A8, A1, A3, A6, A7, and A2

**Figure 9:**
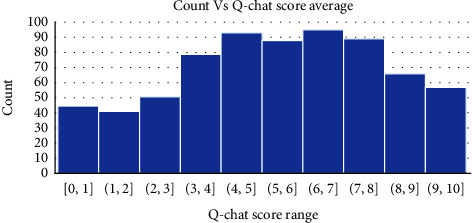
Histogram of Q-chat score for child data.

The feature signatures indicate that all questions of the Q-chat as described in the attribute description section are required for detecting autism. Besides, the authors also tried including the Q-chat score result as a predictor in the data. In that case, the AutoML model selected only that 1 predictor as a feature signature. The result is depicted in Figure 9.

**Figure 10:**
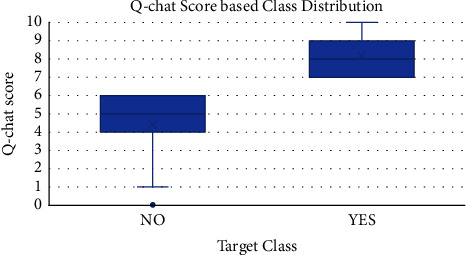
Class distribution based on Q-chat score—the higher the score, the higher the probability of “yes.”

The graph and histogram are shown for the class probability “Yes” which indicates the probability of autism based on the Q-chat score. When the score value is above a certain threshold (∼30%), all subjects are classified as autistic. This is valid proof that the AutoML model is robust in its performance and auto updates itself as per the changes in the training dataset. The graph showing the class distribution based on the Q-chat score is portrayed in Figure 10.

The authors also elaborate on the difference between the feature selection, aggressive feature selection, and the basic classification model. Feature selection works by repeated epochs where the focus is placed by the AutoML model only on achieving a high-performance metric.

Aggressive feature selection concentrates more on the minimal signature size and reveals the most reduced signature with a reasonable performance above a certain threshold as decided by the AutoML model itself. Feature selection is crucial to this study as the proposed work has identified that ethnicity/jaundice/exposure to app/country of residence are not potential nonclinical markers for autism, especially during the infancy stages and early years of growth.

Besides, the authors confirm the effectiveness of the signature markers identified by the AutoML hybrid technique for feature selection and classification by visualizing the projection of the data using principal component analysis [32, 47] and recording the planes that retained most of the original data distribution. This information is available in the Supplementary files S1–S4 and S5–S8 for the child and toddler datasets, respectively. The PCA projects the data using the information contained in the feature signatures and can project ∼95% of the data on the lower dimensional plane. The principal component values and the corresponding density plots that are projected for the respective target classes are available as Supplementary files for both the toddler and child datasets.

Probability values should be close to 1 for the positive class and close to 0 for the negative class when plotted according to the predictors with high performance [47]. It is inferred from the present study that the model selected by AutoML (SES + SVM) can distinguish with precise probability the correct class of data samples for the child dataset. The model, however, reveals minor overlaps for the toddler data. This is attributed to the relative class imbalance in the dataset, which may be resolved by augmenting the data with more samples to balance the class ratio. The exact probability values for each of the samples using the feature signature generated are provided as supplementary files for both the toddler and child datasets.

**Figure 11:**
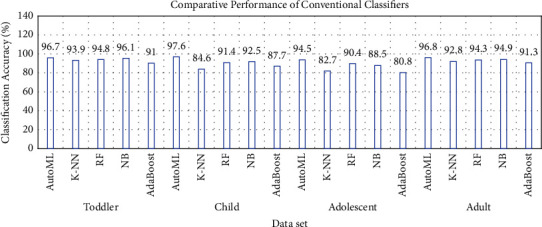
Comparative performance of traditional classifiers on autism datasets.

The authors also explored the performance of traditional learning models on all four datasets to further establish the superior performance of AutoML models in terms of time, performance, and minimal expert inputs. The comparative performance using the ORANGE data mining tool [48] is depicted in Table 5. The results are shown as a table and a graph. The graph portrayed in Figure 11 displays the autism classification accuracy of K-nearest neighbor (K-NN), naïve Bayes classifier (NB), random forest (RF), and AdaBoost algorithms [49] on the four datasets with all the features.

It is to be recorded here that the previous work has reported the accuracy using different performance evaluation methods like train-test ratio, cross-validation, and real-time test datasets. This result is reported using the standard 10-fold cross-validation method and clearly reveals the higher performance of AutoML models in classification. Furthermore, the authors also highlight the role of feature signatures in classification using conventional learning methods. The FCBF (fast correlation-based filter) algorithm uses the idea of identifying features that are highly indicative of the target and highly independent of the other variables as well. The algorithm relies on a metric called Symmetrical Uncertainty (SU) that uses information theory [50]. The difference here is that the authors need to choose the number of features to be ranked and these features are to be given to the classifier. The FCBF algorithm generated slightly different feature signatures than the ones chosen by the AutoML models. The results are tabulated in Table 6.

It is evident from the results in Table 6 that the feature signatures generated by AutoML models, aid in improving classification accuracy. The authors aim to extend this work to biosignature detection for ASD and computationally identify genetic variants/mutants that could aid in autism therapy and study the efficiency of AutoML on a multiclass categorization of neuro-developmental disorders. This work will also be expanded to evaluate the role of feature construction methods on genetic/image data for autism classification and assess the performance of AutoML models on autism classification with the new features generated.

## 6. Conclusion

Research in the field of autism and its causes, symptomatic variations, and therapy has been one of the extensively researched spheres in the recent past. Owing to the adverse effects caused by this neurodevelopmental condition, parents and caretakers are keen to ascertain the predictive features of this disorder. Machine learning and computational intelligence have influenced several healthcare areas providing solutions in the form of predictive and diagnostic models, computer-aided assistantships to physicians, and recommender systems for therapeutic guidance. This research work fuses the competence of automated machine learning and computational intelligence to discover highly predictive features for autism that would enable possible early detection of the disorder. As the globe soars towards automated solutions for everyday living, exploring the possibility of automating the diagnostic procedure for diseases through Artificial Intelligence solutions is a great step forward in the field of science and technology. The authors believe the work undertaken in this research provides an informative insight into autism prevalence irrespective of the subject's ethnic, social, or family status. The authors in the future would focus on biosignature detection for autism based on genomic data and the possibility of automatically extracting image-based feature signatures for autism diagnosis.

## Figures and Tables

**Table 1:** Summary of research findings from the literature on ASD classification using machine learning models.

Year	Data	Model	Features	Tool	Accuracy (%)	Evaluation
2019	Adult, adolescent, child—UCI [9]	SVM and RF	All	Matlab	100	90%–10% train-test
2021	Toddler data—Kaggle [24]	Neural network	Not specified	R-studio	99	Test data
2021	Adult, adolescent, child—UCI, and toddler [7]	Stochastic gradient descent, RF, AdaBoost	Not specified	Not mentioned	∼97	Not mentioned
2022	Adult, adolescent, child—UCI [8]	Voting meta classifier	Not specified	Flask web app	∼91–97	Stratified 10-fold cross-validation

^*∗*^SVM-support vector machine; RF-random forest.

**Table 2:** Kaggle toddler dataset description.

Feature	Domain	Description
A1: response_to_name	Binary (0, 1)	Does your child look at you when you call his/her name?
A2: eye_contact	Binary (0, 1)	How easy is it for you to get eye contact with your child?
A3: point_to_objects	Binary (0, 1)	Does your child point to indicate that s/he wants something?
A4: sharing_interest	Binary (0, 1)	Does your child point to share an interest with you?
A5: pretend_play	Binary (0, 1)	Does your child pretend?
A6: follow_looking	Binary (0, 1)	Does your child follow where you are looking?
A7: comfort_someone	Binary (0, 1)	Does your child show signs of wanting to comfort someone upset?
A8: first_words	Binary (0, 1)	Description of child's first words
A9: simple_gesture	Binary (0, 1)	Does your child use simple gestures?
A10: stare_at_nothing	Binary (0, 1)	Does your child stare at nothing with no apparent purpose?
Age	Numeric	Toddlers (months) and others (years)
Score by Q-chat-10	Numeric	1–10 (less than or equal to 3—no ASD traits; >3 ASD traits
Sex	M/F	Male or female
Ethnicity	String	List of common ethnicities in text format
Born with jaundice	Boolean (Y/N)	Whether the case was born with jaundice
Family member with ASD history	Boolean (Y/N)	Whether any immediate family member has a PDD
Who is completing the test	String	Parent, self, caregiver, medical staff, clinician, etc
Class variable (ASD)/target	String	ASD traits or No ASD traits (Yes/No)

**Table 3:** Comparative training estimates of AutoML models on ASD datasets.

Dataset	Data analysis technique	Feature selection method	Classifier	Training metrics (%)		#Features
				∝_MCC_	∝_Bacc_	
Toddler	Built-in	None	SVM, cost = 1; *γ* = 1; polynomial kernel	97.5	98.4	All
	Feature selection (FS)	SES; *α* = 0.05, Max_*k*_ = 2	SVM, cost = 1; *γ* = 1; polynomial kernel	99.2	99.5	9
	Aggressive FS	SES; *α* = 0.05, Max_*k*_ = 2	SVM, linear kernel; cost = 1	90.8	97	9

Child	Built-in	None	Ridge logistic regression; *λ* = 1	94.6	97.6	All
	Feature selection (FS)	SES; *α* = 0.05, Max_k_ = 2	SVM, linear kernel; cost = 1	100	100	10
	*Aggressive FS*	*Both feature selection and aggressive feature selection yielded the same signature*				

Adolescent	Built-in	None	Ridge logistic regression; *λ* = 1	81.0	94.6	All
	Feature selection (FS)	LASSO, penalty = 1	Ridge logistic regression; *λ* = 1	75.5	91.3	12
	Aggressive FS	SES; *α* = 0.05, Max_*k*_ = 2	SVM, radial basis function kernel; cost = 1; *γ* = 1	69.6	86.4	6

Adult	Built-in	None	SVM, radial basis function kernel; cost = 1; *γ* = 1	96.0	99.2	All
	Feature selection (FS)	SES; *α* = 0.05, Max_*k*_ = 2	SVM, linear kernel; cost = 1	91.7	96.5	11
	*Aggressive FS*	*Both feature selection and aggressive feature selection yielded the same signature*				

**Table 4:** Comparative validation estimates of signature generating AutoML models on ASD datasets.

Dataset	AutoML model	Validation metrics					
		Avg_MCC_	Avg_Bacc_	OOS_TP_	OOS_TN_	OOS_FP_	OOS_FN_
Toddler	FS	0.884	0.936	0.309	0.636	0.055	0
	Aggressive FS	0.875	0.96	0.309	0.631	0.06	0

Child	FS	1	1	0.517	0.483	0	0
	Aggressive FS	Same as FS					

Adolescent	FS	0.98	0.988	0.385	0.606	0	0.01
	Aggressive FS	0.864	0.915	0.327	0.606	0	0.067

Adult	FS	Same as aggressive FS					
	Aggressive FS	1	0.929	0.732	0.268	0	0

**Table 5:** Comparative study on the performance of AutoML models with previous work.

Classification model	Dataset—classifier accuracy (%)			
	Toddler	Child	Adolescent	Adult
Neural network [24]	99			
C4.5 [24]	96			
Deep neural network [23]		92		
SGD (stochastic gradient descent) [7]		99.6		
Random forest [9]			97.2	
Soft voting classifier [8]				94.45
Proposed AutoML—all features	**96.7**	**97.6**	**94.5**	**96.8**
Proposed AutoML—Feature selection	**99.6**	**100**	**94.5**	**98.1**
Proposed aggressive feature selective classifier	**95.8**	**100**	**87**	**98.1**

**Table 6:** Performance of conventional machine learning models with feature selection.

S.No	Dataset	Classifier	FCBF feature signature	Accuracy (%)
1	Toddler	K-NN^*∗*^	A9, A5, A6, A7, A4, A1, A2, A8, and A10	95.6
		RF		95.3
		NB		96.6
		AdaBoost		95.2

2	Child	K-NN	A4, A9, A10, A6, A8, A3, A5, A1, A7, and A2	92.5
		RF		91.4
		NB		96.2
		AdaBoost		92.1

(^*∗*^K-NN : K-nearest neighbor, RF-random forest, NB-naïve Bayes).

## Data Availability

All the datasets utilized in this research work are publicly available. Toddler results: https://app.jadbio.com/share/99710d39-0a97-444f-863f-3c338b488c28. Child results: https://app.jadbio.com/share/6148354f-970c-4e4c-9890-99a6e8049ef4. Adolescent results: https://app.jadbio.com/share/5175f291-70af-430e-8e27-aee894477555. Adult results: https://app.jadbio.com/share/e959a4e2-3344-453b-8379-deb46426f681.

## Supplementary Materials

Supplementary Materials
*File S1.* The visualization of the child autism data projection using principal component analysis by recording the planes that retain most of the original data distribution. *File S2*. The variance obtained by principal component analysis for each sample and their projected values on the 2-D plane for the specific target class is recorded on the child autism dataset. *File S3*. The graphical representation of the probability density values for the target class (healthy) in the child autism data is portrayed. *File S4.* The probability density distribution values for each sample and their contribution towards the target classes are recorded for the child autism dataset. *File S5*. The visualization of the toddler autism data projection using principal component analysis by recording the planes that retain most of the original data distribution. *File S6*. The variance obtained by principal component analysis for each sample and their projected values on the 2-D plane for the specific target class is recorded on the toddler autism dataset. *File S7*. The graphical representation of the probability density values for the target class (healthy) in the toddler autism data is portrayed. *File S8*. The probability density distribution values for each sample and their contribution towards the target classes are recorded for the toddler autism dataset.

## References

[1] Alsaade F. W., Alzahrani M. S. (2022). Classification and detection of autism spectrum disorder based on deep learning algorithms. *Computational Intelligence and Neuroscience*.

[2] Khundrakpam B. S., Lewis J. D., Kostopoulos P., Carbonell F., Evans A. C. (2017). Cortical thickness abnormalities in autism spectrum disorders through late childhood, adolescence, and adulthood: a large-scale MRI study. *Cerebral Cortex*.

[3] Misman M., Samah A., Azurah Classification of adults with autism spectrum disorder using deep neural network.

[4] Plitt M., Barnes K. A., Martin A. (2015). Functional connectivity classification of autism identifies highly predictive brain features but falls short of biomarker standards. *NeuroImage: Clinic*.

[5] Zhang Y., Dong Z., Phillips P. (2015). Detection of subjects and brain regions related to Alzheimer’s disease using 3D MRI scans based on eigenbrain and machine learning. *Frontiers in Computational Neuroscience*.

[6] Chaddad A., Li J., Lu Q. (2021). Can autism Be diagnosed with artificial intelligence? A narrative review. *Diagnostics*.

[7] Sujatha R., Chatterjee J. M., Alaboudi A., Jhanjhi N. Z. (2021). A machine learning way to classify autism spectrum disorder. *International Journal of Emerging Technologies in Learning (Online)*.

[8] Bhuvaneshwari R., Mathubaala N., Pranusha S., Lakshmi Harika P., Sumalatha M. R. (2022). Detection of autism spectrum disorder using machine learning. *International Journal of Engineering Research and Technology*.

[9] Erkan U., Thanh D. N. H. (2020). Autism spectrum disorder detection with machine learning methods. *Current Psychiatry Research and Reviews*.

[10] Darweesh A. N., Salem N., Al-Atabany W. (2022). Classification of autism spectrum disorder using convolutional neural network. https://ssrn.com/abstract=4057056.

[11] Megerian J. T., Dey S., Melmed R. D. (2022). Evaluation of an artificial intelligence-based medical device for diagnosis of autism spectrum disorder. *npj Digital Medicine.*.

[12] Silverman M. H., Jedd K., Luciana M. (2015). Neural networks involved in adolescent reward processing: an activation likelihood estimation meta-analysis of functional neuroimaging studies. *NeuroImage*.

[13] Jalaja Jayalakshmi V., Geetha V., Vivek R. (2019). Classification of autism spectrum disorder data using machine learning techniques. *International Journal of Engineering and Advanced Technology (IJEAT) ISSN*.

[14] Jacob S. G., Bait Ali Sulaiman M. M., Bennet B. (2022). Algorithmic approaches to classify autism spectrum disorders: a research perspective. *Procedia Computer Science*.

[15] Gill S., Mouches P., Hu S. (2020). Using machine learning to predict dementia from neuropsychiatric symptom and neuroimaging data. *Journal of Alzheimer’s Disease*.

[16] Park S. M., Jeong B., Oh Da Y. (2021). Identification of major psychiatric disorders from resting-state electroencephalography using a machine learning approach. *Frontiers in Psychiatry*.

[17] Heinsfeld A. S., Franco A. R., Craddock R. C., Buchweitz A., Meneguzzi F. (2018). Identification of autism spectrum disorder using deep learning and the ABIDE dataset. *NeuroImage: Clinic*.

[18] Tamilarasi F. C., Shanmugam J. Convolutional neural network based autism classification.

[19] Papoutsoglou G., Karaglani M., Lagani V. (2021). Automated machine learning optimizes and accelerates predictive modeling from COVID-19 high throughput datasets. *Scientific Reports*.

[20] Gunn S. R. (1998). Support vector machines for classification and regression. *ISIS Tech. Rep.*.

[21] Volkmar F. R., Reichow B., McPartland J. (2012). Classification of autism and related conditions: progress, challenges, and opportunities. Dialogues. *Clinical Neuroscience*.

[22] Ahmed Z. A. T., Aldhyani T. H. H., Jadhav M. E. (2022). Facial features detection system to identify children with autism spectrum disorder: deep learning models. *Computational and Mathematical Methods in Medicine*.

[23] Mohanty A. S., Parida P., Patra K. C. (2021). ASD classification for children using deep neural network. *Global Transitions Proceedings*.

[24] Saihi A., Alshraideh H. (2021). Development of an autism screening classification model for toddlers. https://arxiv.org/abs/2110.01410.

[25] Sherkatghanad Z., Akhondzadeh M., Salari S. (2020). Automated detection of autism spectrum disorder using a convolutional neural network. *Frontiers in Neuroscience*.

[26] Raj S., Masood S. (2020). Analysis and detection of autism spectrum disorder using machine learning techniques. *Procedia Computer Science*.

[27] Ahammed M. S., Niu S., Ahmed M. R., Dong J., Gao X., Chen Y. (2021). DarkASDNet: classification of ASD on functional MRI using deep neural network. *Frontiers in Neuroinformatics*.

[28] Kaggle (2018). ASD Toddler data experiment. https://www.kaggle.com/code/stap20/asd-toddler-data-experiment/data.

[29] Tabtah F. Autism spectrum disorder screening: machine learning adaptation and DSM-5 fulfillment.

[30] Thabtah F. (2017). ASDTests. A mobile app for asd screening. https://www.asdtests.com.

[31] Saleh A. Y., Chern L. H. (2021). Autism spectrum disorder classification using deep learning. *International Journal of Online and Biomedical Engineering (iJOE)*.

[32] Jadbio (2021). Add data: Bio AutoML software suite. https://jadbio.com/%20-%20Just.

[33] Tsamardinos I., Charonyktakis P., Papoutsoglou G. (2022). Just Add Data: automated predictive modeling for knowledge discovery and feature selection. *npj Precision Oncology.*.

[34] Dwivedi S. K., Rawat B. A review paper on data preprocessing: a critical phase in web usage mining process.

[35] Agarwal V. (2015). Research on data preprocessing and categorization technique for smartphone review analysis. *International Journal of Computer Application*.

[36] Khaire U. M., Dhanalakshmi R. Optimizing feature selection parameters using statistically equivalent signature (SES) algorithm.

[37] Muthukrishnan R., Rohini R. LASSO: a feature selection technique in predictive modeling for machine learning.

[38] Mark S. (2017). *Support Vector Machines vs Logistic Regression*.

[39] Maulik D., Zalud I. (2006). *Ultrasound in Obstetrics and Gynecology*.

[40] Alejandro Salazar D., Jorge I., Juan Carlos S. (2012). Comparison between SVM and logistic regression: which one is better to discriminate?. *Revista Colombiana de Estadística*.

[41] Widodo A., Handoyo S. (2017). The classification performance using logistic regression and support vector machine (SVM). *Journal of Theoretical and Applied Information Technology*.

[42] Ramani R. G., Jacob S. G. (2013). Improved classification of lung cancer tumors based on structural and physicochemical properties of proteins using data mining models. *PLoS One*.

[43] Chicco D., Tötsch N., Jurman G. (2021). The Matthews correlation coefficient (MCC) is more reliable than balanced accuracy, bookmaker informedness, and markedness in two-class confusion matrix evaluation. *BioData Mining*.

[44] Chicco D., Jurman G. (2020). The advantages of the Matthews correlation coefficient (MCC) over F1 score and accuracy in binary classification evaluation. *BMC Genomics*.

[45] Munagala N., Saravanan V., Almukhtar F. H., Jhamat N., Kafi N., Khan S. (2022). Supervised approach to identify autism spectrum neurological disorder via label distribution learning. *Computational Intelligence and Neuroscience*.

[46] Wang X., Zhu X., Lin J. (2022). Application of virtual reality technology in adolescent mental health science education. *Wireless Communications and Mobile Computing*.

[47] Hron K., Menafoglio A., Templ M., Hrůzová K., Filzmoser P. (2015). Simplicial principal component analysis for density functions in bayes spaces. *Computational Statistics and Data Analysis*.

[48] Demsar J., Curk T., Erjavec A. (2013). Orange: data mining toolbox in Python. *Journal of Machine Learning Research*.

[49] Alsariera Y. A., Baashar Y., Alkawsi G., Mustafa A., Alkahtani A. A., Ali N. (2022). Assessment and evaluation of different machine learning algorithms for predicting student performance. *Computational Intelligence and Neuroscience*.

[50] Yu L., Liu H. Feature selection for high-dimensional data: a fast correlation-based filter solution.

