# Supplementary material for: Feature Signature Discovery for Autism Detection: An Automated Machine Learning Based Feature Ranking Framework
Source: Comput Intell Neurosci. 2023 Jan 4;2023:6330002. doi: 10.1155/2023/6330002 (PMC9833925; doi:10.1155/2023/6330002)
Supplement: Supplementary Materials — File S1. The visualization of the child autism data projection using principal component analysis by recording the planes that retain most of the original data distribution. File S2. The variance obtained by principal component analysis for each sample and their projected values on the 2-D plane for the specific target class is recorded on the child autism dataset. File S3. The graphical representation of the probability density values for the target class (healthy) in the child autism data is portrayed. File S4. The probability density distribution values for each sample and their contribution towards the target classes are recorded for the child autism dataset. File S5. The visualization of the toddler autism data projection using principal component analysis by recording the planes that retain most of the original data distribution. File S6. The variance obtained by principal component analysis for each sample and their projected values on the 2-D plane for the specific target class is recorded on the toddler autism dataset. File S7. The graphical representation of the probability density values for the target class (healthy) in the toddler autism data is portrayed. File S8. The probability density distribution values for each sample and their contribution towards the target classes are recorded for the toddler autism dataset. [file 6330002.f1.zip › S2-PCA values-Child data.pdf]

Sample	X	Y	Label
sample0	0.444219303	0.43251154	NO
sample1	0.444219303	0.43251154	NO
sample2	0.50027784	0.018204005	NO
sample3	0.821667999	0.525655172	NO
sample4	-1.154719744	-0.08330939	YES
sample5	0.30374439	-0.039406728	NO
sample6	-0.409163279	0.268549572	YES
sample7	-0.464760327	0.084674936	YES
sample8	-0.078139535	0.289503931	YES
sample9	0.31495048	-0.682120517	NO
sample10	-0.244331101	-0.928828318	YES
sample11	1.199236417	0.320997665	NO
sample12	-0.908684203	-0.158802842	YES
sample13	1.4205739	-0.468269	NO
sample14	-1.154719744	-0.08330939	YES
sample15	-1.154719744	-0.08330939	YES
sample16	-1.000021907	0.62340165	YES
sample17	1.666609441	-0.543762452	NO
sample18	-0.621044477	-0.77461139	YES
sample19	-0.565270128	0.755194065	YES
sample20	-0.512891314	0.157711516	YES
sample21	-0.131915506	0.389859121	NO
sample22	0.95229276	0.957549381	NO
sample23	0.008669746	0.670181569	NO
sample24	-0.843915058	0.136757157	YES
sample25	-0.119801299	-0.813912931	NO
sample26	0.009577862	0.109123304	NO
sample27	-1.154719744	-0.08330939	YES
sample28	0.581264496	-0.171727278	NO
sample29	1.1684434	-0.70975437	NO
sample30	-1.154719744	-0.08330939	YES
sample31	0.466369777	0.326033357	NO
sample32	-0.500446854	-0.403961964	YES
sample33	0.345118515	-0.090324864	NO
sample34	0.296987528	-0.017288283	NO
sample35	1.665200428	0.649593081	NO
sample36	1.067823262	0.15236058	NO
sample37	1.419164887	0.725086532	NO
sample38	-1.154719744	-0.08330939	YES
sample39	-0.473932424	-0.027010427	YES
sample40	-0.163127738	0.19305612	NO
sample41	1.665200428	0.649593081	NO
sample42	0.954609888	-0.796864416	NO
sample43	-0.843915058	0.136757157	YES
sample44	1.199737313	-0.311299603	NO
sample45	0.534258333	0.652066759	NO

sample46	0.78946597	0.68825867	NO
sample47	0.124472581	-0.388637331	YES
sample48	-0.457294265	0.341586153	YES
sample49	1.454444054	0.357189575	NO
sample50	1.04453858	-0.385713375	NO
sample51	0.100915566	-0.673081188	NO
sample52	-0.345749018	0.302749076	NO
sample53	-0.768098951	0.121519605	YES
sample54	0.188213888	0.765782073	NO
sample55	1.323141237	-0.00304333	NO
sample56	1.156329193	0.494017683	NO
sample57	-0.998612895	-0.569953882	YES
sample58	-0.743405257	-0.533761972	YES
sample59	-0.457294265	0.341586153	YES
sample60	1.077105696	0.072450122	NO
sample61	-0.998612895	-0.569953882	YES
sample62	0.288222792	0.727560211	NO
sample63	-0.687847616	-0.315772238	YES
sample64	0.179161513	0.35629557	NO
sample65	1.533897611	0.289360175	NO
sample66	1.109307725	-0.090153376	NO
sample67	0.954609888	-0.796864416	NO
sample68	0.356123203	-0.636889277	NO
sample69	-0.998612895	-0.569953882	YES
sample70	0.667200222	-0.670452829	NO
sample71	1.04453858	-0.385713375	NO
sample72	-0.409163279	0.268549572	YES
sample73	-0.843915058	0.136757157	YES
sample74	-0.457294265	0.341586153	YES
sample75	-0.20996005	-0.73566701	NO
sample76	-0.94396337	0.209094116	YES
sample77	0.954609888	-0.796864416	NO
sample78	-0.768098951	0.121519605	YES
sample79	-0.288022355	0.876503614	NO
sample80	0.730831191	0.675562396	NO
sample81	-0.908684203	-0.158802842	YES
sample82	-0.563861116	-0.438161468	YES
sample83	0.534258333	0.652066759	NO
sample84	0.100915566	-0.673081188	NO
sample85	-0.768098951	0.121519605	YES
sample86	-0.179774159	0.209265603	YES
sample87	-1.154719744	-0.08330939	YES
sample88	0.287314676	1.288618476	NO
sample89	-0.908684203	-0.158802842	YES
sample90	-0.611992102	-0.365124887	YES
sample91	0.36537698	-0.323552149	NO
sample92	-0.752577354	-0.645447334	YES

sample93	-0.752577354	-0.645447334	YES
sample94	-1.154719744	-0.08330939	YES
sample95	-0.096988988	-0.670624316	YES
sample96	-0.565270128	0.755194065	YES
sample97	-0.390530533	-0.083137902	YES
sample98	-0.301226824	-0.110943243	YES
sample99	1.067025484	0.521823024	NO
sample100	-0.014725274	0.323703434	NO
sample101	-0.122590798	0.545715525	YES
sample102	1.231857662	-0.675554866	NO
sample103	0.130800466	0.918729112	NO
sample104	0.823077011	-0.66770036	NO
sample105	-0.565270128	0.755194065	YES
sample106	-0.613401115	0.828230645	YES
sample107	1.109307725	-0.090153376	NO
sample108	0.746313381	-0.057289447	NO
sample109	1.0679336	-0.039235241	NO
sample110	-0.096364006	-0.231667935	YES
sample111	1.16395725	0.688894622	NO
sample112	-0.597879517	0.061263706	YES
sample113	-0.899512106	-0.047117479	YES
sample114	0.057708849	0.036086723	NO
sample115	-1.154719744	-0.08330939	YES
sample116	-0.768098951	0.121519605	YES
sample117	-0.115235074	0.994222563	NO
sample118	-0.20996005	-0.73566701	NO
sample119	0.911826751	0.447409252	NO
sample120	-0.543229992	0.840311703	YES
sample121	0.090955075	-0.521509426	NO
sample122	-1.154719744	-0.08330939	YES
sample123	0.458903715	0.06912214	NO
sample124	-0.477091242	-0.023368733	YES
sample125	-0.441812075	-0.39126569	YES
sample126	-0.354513754	1.04759757	YES
sample127	-0.285705226	-0.877910183	YES
sample128	0.158218649	-0.634432406	NO
sample129	-0.353104741	-0.145757962	YES
sample130	-0.768098951	0.121519605	YES
sample131	1.009259413	-0.017816418	NO
sample132	0.067386205	0.884529608	NO
sample133	0.441795805	0.683513794	NO
sample134	-1.000021907	0.62340165	YES
sample135	0.265410481	0.584271596	NO
sample136	0.076341594	-0.315600751	YES
sample137	1.821307277	0.162948588	NO
sample138	0.248147341	-0.223281365	NO
sample139	-1.154719744	-0.08330939	YES

sample140	-0.621044477	-0.77461139	YES
sample141	0.203696078	0.03293023	NO
sample142	-0.409163279	0.268549572	YES
sample143	-0.353104741	-0.145757962	YES
sample144	0.209270131	0.163092344	NO
sample145	-0.768098951	0.121519605	YES
sample146	0.757128914	-0.259301788	NO
sample147	-0.768098951	0.121519605	YES
sample148	-0.131007389	-0.171199143	NO
sample149	1.078284649	-0.631508449	NO
sample150	-0.178649336	0.96002306	YES
sample151	-0.37704293	-0.095705691	YES
sample152	0.146512717	-0.303519693	NO
sample153	-0.768098951	0.121519605	YES
sample154	-0.390530533	-0.083137902	YES
sample155	-0.719967965	0.048483025	YES
sample156	0.056353965	-0.225273772	NO
sample157	-0.40043795	-0.442183825	YES
sample158	-0.234653744	-0.080385433	YES
sample159	-1.154719744	-0.08330939	YES
sample160	-0.409163279	0.268549572	YES
sample161	-0.244331101	-0.928828318	YES
sample162	1.032424373	0.818058678	NO
sample163	-0.324582436	-0.491536474	YES
sample164	0.746423719	-0.248885268	NO
sample165	0.577041471	-0.592206908	NO
sample166	0.814024637	-1.077186863	NO
sample167	-0.563861116	-0.438161468	YES
sample168	-0.753986366	0.547908198	YES
sample169	-0.998612895	-0.569953882	YES
sample170	0.76630101	-0.147616426	NO
sample171	0.913235763	-0.745946281	NO
sample172	-1.154719744	-0.08330939	YES
sample173	0.223493054	0.397885116	NO
sample174	0.458903715	0.06912214	NO
sample175	0.699537278	0.277107629	NO
sample176	0.610233569	0.30491297	NO
sample177	-1.154719744	-0.08330939	YES
sample178	0.286907316	0.432084619	NO
sample179	-0.285705226	-0.877910183	YES
sample180	-0.613401115	0.828230645	YES
sample181	1.012336551	-0.223109877	NO
sample182	-0.711203228	-0.696365469	YES
sample183	0.278142579	1.176933113	NO
sample184	-0.409163279	0.268549572	YES
sample185	0.232448443	-0.802245243	NO
sample186	-0.310279198	-0.520429746	YES

sample187	-1.154719744	-0.08330939	YES
sample188	-0.062617937	-0.477463009	NO
sample189	-0.777151326	-0.287966897	YES
sample190	-0.098398	0.522731216	YES
sample191	-0.045937506	0.126900433	YES
sample192	-0.146528987	0.595767797	YES
sample193	0.110087663	-0.561395825	NO
sample194	0.676142259	-0.069370505	NO
sample195	1.131755221	0.851498119	NO
sample196	-0.613401115	0.828230645	YES
sample197	-0.285705226	-0.877910183	YES
sample198	-0.908684203	-0.158802842	YES
sample199	0.954609888	-0.796864416	NO
sample200	0.954609888	-0.796864416	NO
sample201	-0.512891314	0.157711516	YES
sample202	0.913235763	-0.745946281	NO
sample203	-0.178649336	0.96002306	YES
sample204	-0.621044477	-0.77461139	YES
sample205	0.287314676	1.288618476	NO
sample206	-0.74481427	0.659593561	YES
sample207	-0.94396337	0.209094116	YES
sample208	1.109307725	-0.090153376	NO
sample209	-0.153955642	0.304741483	NO
sample210	-0.409163279	0.268549572	YES
sample211	0.232448443	-0.802245243	NO
sample212	0.534258333	0.652066759	NO
sample213	0.100915566	-0.673081188	NO
sample214	-0.377951047	0.465352573	NO
sample215	-1.154719744	-0.08330939	YES
sample216	-0.743405257	-0.533761972	YES
sample217	1.56609964	0.126756677	NO
sample218	0.944447995	-0.549143279	NO
sample219	-0.998612895	-0.569953882	YES
sample220	-1.154719744	-0.08330939	YES
sample221	-0.333347172	0.25331202	YES
sample222	-0.08719191	-0.119982572	YES
sample223	-0.613401115	0.828230645	YES
sample224	0.610233569	0.30491297	NO
sample225	-1.154719744	-0.08330939	YES
sample226	0.591154056	-0.165818315	NO
sample227	-0.285705226	-0.877910183	YES
sample228	0.823196733	-0.9655015	NO
sample229	-0.789265533	0.915805155	YES
sample230	-0.711203228	-0.696365469	YES
sample231	0.59031687	0.237759225	NO
sample232	-0.087311631	0.177818568	YES
sample233	-0.753986366	0.547908198	YES

sample234	0.634768133	-0.01845237	NO
sample235	-0.163127738	0.19305612	NO
sample236	-0.768098951	0.121519605	YES
sample237	0.310262928	0.81267785	NO
sample238	-0.09054138	0.338940986	NO
sample239	0.26486718	0.34696698	NO
sample240	0.075433478	0.245457514	NO
sample241	0.289631804	-0.465795321	NO
sample242	1.255294954	-0.09330987	NO
sample243	1.264467051	0.018375493	NO
sample244	0.14763754	0.447237764	NO
sample245	0.112358374	0.815134721	NO
sample246	0.920998847	0.559094614	NO
sample247	1.666609441	-0.543762452	NO
sample248	1.123992137	-0.453542775	NO
sample249	0.19104566	-0.358079522	NO
sample250	-0.186522758	-0.153422014	YES
sample251	-0.473932424	-0.027010427	YES
sample252	-0.096988988	-0.670624316	YES
sample253	-0.563861116	-0.438161468	YES
sample254	0.498979166	1.019963716	NO
sample255	0.543430429	0.763752122	NO
sample256	0.510902721	0.271473529	NO
sample257	-1.154719744	-0.08330939	YES
sample258	0.954609888	-0.796864416	NO
sample259	-0.563861116	-0.438161468	YES
sample260	0.032107038	1.252426565	NO
sample261	0.667089884	-0.478857008	NO
sample262	-1.154719744	-0.08330939	YES
sample263	0.155565091	0.10596681	NO
sample264	-0.611992102	-0.365124887	YES
sample265	-0.789265533	0.915805155	YES
sample266	0.575632458	0.601148624	NO
sample267	-0.998612895	-0.569953882	YES
sample268	-0.145119975	-0.597587736	YES
sample269	0.746543441	-0.546686408	NO
sample270	-0.319234587	0.679700613	YES
sample271	0.853110291	0.233061212	NO
sample272	0.944447995	-0.549143279	NO
sample273	-0.777151326	-0.287966897	YES
sample274	-0.768098951	0.121519605	YES
sample275	0.386238164	0.465524061	NO
sample276	0.149046553	-0.746117768	NO
sample277	-0.146528987	0.595767797	YES
sample278	0.722686933	-0.294982371	NO
sample279	-0.899512106	-0.047117479	YES
sample280	1.278689974	0.253168264	NO

sample281	0.067386205	0.884529608	NO
sample282	-0.076357865	0.607848855	NO
sample283	0.64353287	-0.763300864	NO
sample284	0.289631804	-0.465795321	NO
sample285	-0.621274537	-0.285214429	YES
sample286	-0.908684203	-0.158802842	YES
sample287	-1.154719744	-0.08330939	YES
sample288	0.954609888	-0.796864416	NO
sample289	-0.177240323	-0.233332472	YES
sample290	-0.687847616	-0.315772238	YES
sample291	1.1684434	-0.70975437	NO
