# Supplementary material for: Feature Signature Discovery for Autism Detection: An Automated Machine Learning Based Feature Ranking Framework
Source: Comput Intell Neurosci. 2023 Jan 4;2023:6330002. doi: 10.1155/2023/6330002 (PMC9833925; doi:10.1155/2023/6330002)
Supplement: Supplementary Materials — File S1. The visualization of the child autism data projection using principal component analysis by recording the planes that retain most of the original data distribution. File S2. The variance obtained by principal component analysis for each sample and their projected values on the 2-D plane for the specific target class is recorded on the child autism dataset. File S3. The graphical representation of the probability density values for the target class (healthy) in the child autism data is portrayed. File S4. The probability density distribution values for each sample and their contribution towards the target classes are recorded for the child autism dataset. File S5. The visualization of the toddler autism data projection using principal component analysis by recording the planes that retain most of the original data distribution. File S6. The variance obtained by principal component analysis for each sample and their projected values on the 2-D plane for the specific target class is recorded on the toddler autism dataset. File S7. The graphical representation of the probability density values for the target class (healthy) in the toddler autism data is portrayed. File S8. The probability density distribution values for each sample and their contribution towards the target classes are recorded for the toddler autism dataset. [file 6330002.f1.zip › S4-Density Distribution Values-Child data.pdf]

Class "NO"	Class "YES"
0.999071107	3.00E-14
0.99906731	0.082986713
0.9990806	2.05E-06
0.999991682	0.082682831
0.999087574	0.082363468
0.9990863	1.68E-10
0.9999999	3.00E-14
0.9999999	3.00E-14
0.9999999	1.63E-10
0.99909195	2.01E-06
0.9999999	2.02E-06
0.999090275	2.03E-06
0.908334748	2.04E-06
0.908652359	3.00E-14
0.999096017	3.00E-14
0.9999999	0.08387111
0.999991757	3.00E-14
0.999070998	2.04E-06
0.999075938	2.03E-06
0.9999999	0.082355233
0.9999999	0.082918616
0.9999999	1.70E-10
0.908942861	1.67E-10
0.9999999	2.02E-06
0.999991671	0.083130327
0.9999999	1.68E-10
0.999094861	1.66E-10
0.999991684	1.67E-10
0.9999999	0.082987705
0.99999167	2.04E-06
0.908907229	0.083218217
0.907514976	1.70E-10
0.999092995	1.68E-10
0.9999999	1.70E-10
0.9999999	2.04E-06
0.9999999	1.68E-10
0.908606555	0.082780978
0.999088218	3.00E-14
0.9999999	1.69E-10
0.9999999	2.16E-06
0.999991613	2.06E-06
0.999095719	2.12E-06
0.999093401	3.00E-14
0.999991656	0.083783367
0.908363648	1.94E-06
0.999991649	2.02E-06

0.908312066	2.02E-06
0.9999999	0.083284526
0.999088041	1.99E-06
0.909132162	2.05E-06
0.999088571	0.080271262
0.999087407	0.08298038
0.9999999	1.66E-10
0.910692802	3.00E-14
0.9999999	1.68E-10
0.99908805	0.083926636
0.999991774	3.00E-14
0.9999999	2.02E-06
0.999991664	2.06E-06
0.9999999	0.083077177
0.9999999	0.084571977
0.908054348	0.083297464
0.907113745	1.68E-10
0.908866132	1.67E-10
0.999991676	0.082360713
0.999087717	3.00E-14
0.999087883	2.02E-06
0.907955392	0.083134472
0.999991666	0.083510041
0.909603594	1.68E-10
0.999084683	1.68E-10
0.999088063	0.083398968
0.9999999	0.082262986
0.908378144	1.68E-10
0.908879005	2.00E-06
0.908541918	1.69E-10
0.999090316	1.98E-06
0.90876187	0.082472297
0.9999999	3.00E-14
0.90896843	0.08281254
0.908106016	0.08206933
0.9999999	0.082906805
0.999991751	2.04E-06
0.999099221	2.00E-06
0.99999175	1.67E-10
0.999083007	3.00E-14
0.999991739	3.00E-14
0.999095031	0.084571977
0.999087883	2.03E-06
0.99999171	2.02E-06
0.999991603	0.082832653
0.999991743	2.00E-06
0.999991691	3.00E-14

0.999092599	1.66E-10
0.908401008	0.082549908
0.908750935	0.083043418
0.909460881	0.081821417
0.999080066	2.05E-06
0.9999999	0.082824863
0.999991649	1.69E-10
0.999991404	2.03E-06
0.99999171	0.082987816
0.999088571	2.02E-06
0.9999999	2.02E-06
0.908913518	1.69E-10
0.908465806	0.082987705
0.999088041	3.00E-14
0.908907229	2.14E-06
0.90894933	1.68E-10
0.9999999	3.00E-14
0.9999999	2.04E-06
0.999991625	0.082045875
0.999991723	1.98E-06
0.999991605	3.00E-14
0.999091158	0.082618805
0.999084372	2.04E-06
0.909320048	2.04E-06
0.999087046	0.083690597
0.99907547	2.04E-06
0.999086376	1.68E-10
0.909267958	0.080847695
0.909056165	2.06E-06
0.9999999	0.082772283
0.9999999	2.04E-06
0.907686939	3.00E-14
0.908504124	2.04E-06
0.999991498	3.00E-14
0.9999999	2.05E-06
0.9999999	2.03E-06
0.903488552	1.72E-10
0.999087216	0.082824863
0.999082764	0.083006013
0.999991651	1.65E-10
0.99999166	1.68E-10
0.909144886	0.082621469
0.999074269	1.67E-10
0.909538061	2.02E-06
0.999083753	1.68E-10
0.999991808	3.00E-14
0.9999999	0.083743735

0.9999999	1.66E-10
0.999991736	
0.910234745	
0.999991614	
0.9999999	
0.908817798	
0.908728582	
0.999991742	
0.910924852	
0.999991613	
0.9999999	
