# Supplementary material for: Feature Signature Discovery for Autism Detection: An Automated Machine Learning Based Feature Ranking Framework
Source: Comput Intell Neurosci. 2023 Jan 4;2023:6330002. doi: 10.1155/2023/6330002 (PMC9833925; doi:10.1155/2023/6330002)
Supplement: Supplementary Materials — File S1. The visualization of the child autism data projection using principal component analysis by recording the planes that retain most of the original data distribution. File S2. The variance obtained by principal component analysis for each sample and their projected values on the 2-D plane for the specific target class is recorded on the child autism dataset. File S3. The graphical representation of the probability density values for the target class (healthy) in the child autism data is portrayed. File S4. The probability density distribution values for each sample and their contribution towards the target classes are recorded for the child autism dataset. File S5. The visualization of the toddler autism data projection using principal component analysis by recording the planes that retain most of the original data distribution. File S6. The variance obtained by principal component analysis for each sample and their projected values on the 2-D plane for the specific target class is recorded on the toddler autism dataset. File S7. The graphical representation of the probability density values for the target class (healthy) in the toddler autism data is portrayed. File S8. The probability density distribution values for each sample and their contribution towards the target classes are recorded for the toddler autism dataset. [file 6330002.f1.zip › S6-PCA values - Toddler data.pdf]

Sample	X	Y	Label
1	0.967317345	-0.00683555	No
3	0.643896329	-0.491563311	Yes
4	-1.426282315	0.05832638	Yes
5	-1.110710939	-0.421519617	Yes
8	-0.073513606	-0.28944967	Yes
9	1.246772172	-0.050379165	No
10	-0.758850659	-0.206793486	Yes
11	-0.167567243	-0.070915939	Yes
12	-0.692509064	-0.173286342	Yes
13	1.561786287	0.131371616	No
14	-0.428837543	-0.01108395	Yes
16	-0.335363915	-0.405864608	Yes
17	1.561786287	0.131371616	No
18	-0.696722692	-0.500077441	Yes
19	0.881734103	-0.233276597	No
20	-0.335363915	-0.405864608	Yes
21	-0.803653238	0.092707224	Yes
22	-0.839769787	0.529009606	Yes
23	-0.447579331	0.634224455	Yes
25	-1.426282315	0.05832638	Yes
26	1.561786287	0.131371616	No
27	-0.733568856	-0.533052791	Yes
28	-1.426282315	0.05832638	Yes
29	-0.434444822	1.151493815	Yes
30	1.561786287	0.131371616	No
31	1.561786287	0.131371616	No
32	1.561786287	0.131371616	No
33	1.561786287	0.131371616	No
34	1.561786287	0.131371616	No
36	-1.426282315	0.05832638	Yes
37	1.561786287	0.131371616	No
39	1.561786287	0.131371616	No
41	1.561786287	0.131371616	No
42	1.561786287	0.131371616	No
43	1.561786287	0.131371616	No
45	1.561786287	0.131371616	No
46	1.246772172	-0.050379165	No
48	1.561786287	0.131371616	No
49	1.561786287	0.131371616	No
51	1.561786287	0.131371616	No
53	1.561786287	0.131371616	No
54	-1.426282315	0.05832638	Yes
55	1.561786287	0.131371616	No
57	0.184850298	-0.517786586	Yes
61	1.561786287	0.131371616	No
63	-0.758293398	-0.868390264	Yes

64	-0.139206688	0.902204193	Yes
65	0.516655288	1.169621434	No
66	-1.146827488	0.014782764	Yes
67	0.610686177	0.113243998	Yes
68	1.28233146	0.174915231	No
69	0.922793896	0.126489852	No
70	-0.839769787	0.529009606	Yes
71	-0.381151316	-0.979923438	Yes
72	-0.516348771	1.013737367	Yes
73	-1.426282315	0.05832638	Yes
75	0.259662287	-0.92925422	Yes
76	-0.803653238	0.092707224	Yes
77	1.561786287	0.131371616	No
78	1.561786287	0.131371616	No
80	0.958910444	-0.30981253	No
81	1.254728587	-0.382855226	No
82	-0.762593446	0.452473674	Yes
83	-0.733568856	-0.533052791	Yes
85	-0.097482861	-0.696675954	Yes
86	0.11423672	0.715642151	Yes
87	1.561786287	0.131371616	No
88	0.894354632	0.396491482	No
90	-1.426282315	0.05832638	Yes
91	1.561786287	0.131371616	No
93	-0.758293398	-0.868390264	Yes
94	1.28233146	0.174915231	No
95	0.237757722	0.551568271	Yes
96	1.254728587	-0.382855226	No
97	-0.839769787	0.529009606	Yes
99	1.561786287	0.131371616	No
100	1.561786287	0.131371616	No
101	0.893797371	1.05808826	No
102	0.605958569	-0.101048383	Yes
103	0.20957484	-0.182449113	Yes
104	0.274710661	-0.712506197	Yes
105	0.569948795	-0.96179582	Yes
106	-1.426282315	0.05832638	Yes
107	1.561786287	0.131371616	No
109	-0.381151316	-0.979923438	Yes
110	1.561786287	0.131371616	No
112	0.660259644	-0.521062391	Yes
113	0.967317345	-0.00683555	No
115	-0.478838571	-0.91193388	Yes
116	-0.795803598	1.057280982	Yes
117	0.578783256	0.876337479	Yes
118	-1.426282315	0.05832638	Yes
121	0.230665763	0.002344188	Yes

123	1.28233146	0.174915231	No
124	1.200427511	0.037158782	No
125	-0.831256112	-0.465063233	Yes
129	-0.139206688	0.902204193	Yes
130	-0.454114029	-0.576596407	Yes
131	-0.417267864	-0.543621056	Yes
133	1.561786287	0.131371616	No
134	0.881734103	-0.233276597	No
135	0.295221575	-0.703959824	Yes
136	1.205155119	0.251451163	No
137	-1.426282315	0.05832638	Yes
139	-1.426282315	0.05832638	Yes
140	0.274710661	-0.712506197	Yes
141	-1.426282315	0.05832638	Yes
142	-0.469897336	-0.370850399	Yes
143	0.290493968	-0.918252205	Yes
144	0.939714472	-0.564606007	No
146	-1.426282315	0.05832638	Yes
147	-0.795696824	-0.239768836	Yes
148	1.561786287	0.131371616	No
150	0.958910444	-0.30981253	No
152	0.587296931	-0.11773536	Yes
153	0.894354632	0.396491482	No
154	-1.426282315	0.05832638	Yes
156	-1.110710939	-0.421519617	Yes
157	-1.146827488	0.014782764	Yes
159	-0.054073933	0.4931922	Yes
160	-0.746230131	0.422974593	Yes
161	-0.787289923	0.063208143	Yes
162	-0.524755672	0.710760387	Yes
163	-1.146827488	0.014782764	Yes
166	1.561786287	0.131371616	No
167	0.939714472	-0.564606007	No
168	1.246772172	-0.050379165	No
169	1.561786287	0.131371616	No
171	0.491378803	0.604108977	No
172	-0.462047696	0.593723359	Yes
173	1.561786287	0.131371616	No
174	1.254728587	-0.382855226	No
175	1.254728587	-0.382855226	No
176	0.2409205	-0.283945816	Yes
177	0.889583743	0.73129716	No
178	0.884856136	0.517004779	No
179	0.56143512	0.032277018	No
181	-0.053516672	-0.168404578	Yes
182	1.209368747	0.578242263	No
183	0.499864413	-0.336035805	No

184	0.570376355	0.573360499	No
186	0.512998922	0.181233556	Yes
187	-1.119224614	0.572553221	Yes
189	1.254728587	-0.382855226	No
190	0.931307571	-0.867582987	No
192	0.967317345	-0.00683555	No
193	0.616293456	-1.049333768	Yes
194	1.209368747	0.578242263	No
196	-0.790196319	-0.105296783	Yes
197	-0.474624944	-0.58514278	Yes
200	0.267511928	0.035319538	Yes
201	-1.049140232	-0.053206794	Yes
202	-1.069651147	-0.061753168	Yes
203	-0.462627704	0.417476432	Yes
205	-0.692509064	-0.173286342	Yes
206	0.616293456	-1.049333768	Yes
208	-0.10309014	0.465901811	Yes
209	0.54095227	-0.030197412	No
210	1.205155119	0.251451163	No
211	0.58673967	0.543861418	No
212	-0.758293398	-0.868390264	Yes
213	-0.434872382	-0.383662503	Yes
214	-1.426282315	0.05832638	Yes
216	0.92991392	0.621785878	No
217	0.823285429	0.148691956	No
218	-1.146827488	0.014782764	Yes
219	-0.41014784	-0.04832503	Yes
220	-0.725719216	0.431520967	Yes
221	-0.428837543	-0.01108395	Yes
222	1.561786287	0.131371616	No
224	-1.426282315	0.05832638	Yes
225	-1.049140232	-0.053206794	Yes
226	0.610686177	0.113243998	Yes
230	-0.17178087	-0.397707038	Yes
231	0.184850298	-0.517786586	Yes
232	-1.119224614	0.572553221	Yes
233	-1.426282315	0.05832638	Yes
234	0.168537795	0.295628143	Yes
235	-0.10309014	0.465901811	Yes
236	1.246772172	-0.050379165	No
237	-0.331150288	-0.079073508	Yes
238	1.561786287	0.131371616	No
240	0.605958569	-0.101048383	No
241	-1.426282315	0.05832638	Yes
242	-0.787847184	0.724804921	Yes
243	-0.804210499	0.754304002	Yes
245	-0.479395832	-0.250337102	Yes

246	-0.741502523	0.637266974	Yes
247	-0.209184296	0.23091439	Yes
248	-0.717233606	-0.508623815	Yes
249	-1.426282315	0.05832638	Yes
250	0.843796343	0.15723833	No
251	-1.110710939	-0.421519617	Yes
252	0.233544095	0.224777172	Yes
253	-0.480232222	0.577434985	Yes
255	-1.049140232	-0.053206794	Yes
256	-1.049140232	-0.053206794	Yes
257	-0.757865838	0.666766055	Yes
258	0.97527376	-0.33931161	No
259	-0.478838571	-0.91193388	Yes
260	0.56199238	-0.62931976	No
261	-0.437778779	-0.55216743	Yes
262	-1.426282315	0.05832638	Yes
263	-0.168124504	0.590680839	Yes
268	-0.0577303	-0.495195677	Yes
269	-0.10309014	0.465901811	Yes
271	1.561786287	0.131371616	No
272	0.263875915	-0.602463121	Yes
273	0.56143512	0.032277018	No
274	0.56143512	0.032277018	No
276	-0.122207403	-1.032013427	Yes
277	-0.787289923	0.063208143	Yes
278	-0.472275808	0.244958924	Yes
279	1.561786287	0.131371616	No
280	-1.426282315	0.05832638	Yes
281	-1.426282315	0.05832638	Yes
283	-0.454114029	-0.576596407	Yes
284	-1.146827488	0.014782764	Yes
285	-0.758293398	-0.868390264	Yes
286	0.931307571	-0.867582987	No
289	0.239151373	-0.937800594	Yes
293	0.155853773	0.413811822	Yes
294	-0.40166223	-0.988469812	Yes
295	0.221724527	-0.538739293	Yes
297	0.881734103	-0.233276597	No
298	-1.069651147	-0.061753168	Yes
299	-1.146827488	0.014782764	Yes
300	-0.692509064	-0.173286342	Yes
303	-0.749352163	-0.327306784	Yes
304	-1.146827488	0.014782764	Yes
305	-0.762593446	0.452473674	Yes
306	1.28233146	0.174915231	No
307	0.151640146	0.087020723	Yes
308	-1.426282315	0.05832638	Yes

309	-0.101696489	-1.023467053	Yes
310	-0.118037056	-0.156124268	Yes
311	-0.127814681	0.130564338	Yes
313	-1.426282315	0.05832638	Yes
314	1.561786287	0.131371616	No
316	-0.082027281	0.704623169	Yes
317	-0.516348771	1.013737367	Yes
318	-0.430658755	-0.056871404	Yes
319	0.299435203	-0.377168724	Yes
320	1.561786287	0.131371616	No
321	1.561786287	0.131371616	No
322	-0.155417555	-0.427206119	Yes
323	-1.426282315	0.05832638	Yes
324	-1.426282315	0.05832638	Yes
325	0.259662287	-0.92925422	Yes
326	-0.122207403	-1.032013427	Yes
327	0.524588955	-0.000698332	No
328	-0.787847184	0.724804921	Yes
329	1.561786287	0.131371616	No
330	-0.101268928	0.511689265	Yes
332	-0.093312514	0.179213205	Yes
333	-0.462047696	0.593723359	Yes
334	0.282537553	-0.585776144	Yes
337	0.92991392	0.621785878	No
338	1.209368747	0.578242263	No
339	-1.426282315	0.05832638	Yes
340	-1.119224614	0.572553221	Yes
342	-0.443279283	-0.686639483	Yes
343	-1.426282315	0.05832638	Yes
345	-0.434872382	-0.383662503	Yes
346	-0.127814681	0.130564338	Yes
347	-0.01979254	-0.885710605	Yes
349	0.201213613	-0.547285666	Yes
350	1.561786287	0.131371616	No
351	1.561786287	0.131371616	No
352	1.561786287	0.131371616	No
353	1.561786287	0.131371616	No
354	-0.427068416	0.642770828	Yes
356	-1.049140232	-0.053206794	Yes
357	-0.478838571	-0.91193388	Yes
358	-0.831256112	-0.465063233	Yes
360	1.561786287	0.131371616	No
361	0.176364687	0.422358196	Yes
363	-0.163396896	0.80497322	Yes
364	-0.381151316	-0.979923438	Yes
365	-1.049140232	-0.053206794	Yes
366	0.893797371	1.05808826	No

367	-1.1112682	0.240077161	Yes
368	-1.1112682	0.240077161	Yes
369	-0.40166223	-0.988469812	Yes
370	-0.097482861	-0.696675954	Yes
373	-0.154989995	1.1079502	Yes
374	-0.201334656	1.195488148	Yes
375	1.200427511	0.037158782	No
376	0.537723463	0.516571029	Yes
378	-0.524198411	0.049163609	Yes
379	0.517212549	0.508024656	Yes
381	0.56199238	-0.62931976	Yes
382	-1.069651147	-0.061753168	Yes
383	1.561786287	0.131371616	No
384	1.200427511	0.037158782	No
385	0.885413396	-0.144591999	No
386	0.532995856	0.302278648	Yes
387	-0.443279283	-0.686639483	Yes
389	0.890141004	0.069700382	No
392	0.336838628	-1.005790152	Yes
394	0.605958569	-0.101048383	Yes
395	-1.110710939	-0.421519617	Yes
396	1.561786287	0.131371616	No
397	0.200656352	0.114311112	Yes
399	0.528782228	-0.024512451	Yes
400	-0.524198411	0.049163609	Yes
401	-1.049140232	-0.053206794	Yes
402	1.254728587	-0.382855226	No
403	-0.831256112	-0.465063233	Yes
404	-1.1112682	0.240077161	Yes
405	-0.795803598	1.057280982	Yes
406	-0.795803598	1.057280982	Yes
407	1.561786287	0.131371616	No
409	0.614899804	0.440035097	Yes
410	-1.146827488	0.014782764	Yes
411	-1.426282315	0.05832638	Yes
412	0.249327401	0.019031164	Yes
413	-0.060636696	-0.663700604	Yes
414	1.254728587	-0.382855226	No
415	0.651852744	-0.824039371	Yes
416	1.561786287	0.131371616	No
418	-0.074093615	-0.465696597	Yes
419	0.590175263	0.104697624	No
420	-0.758293398	-0.868390264	Yes
421	-0.209184296	0.23091439	Yes
422	-0.123601054	0.457355437	Yes
423	0.610128916	0.774840776	No
425	-0.392720995	-0.447386331	Yes

427	0.282537553	-0.585776144	Yes
428	0.546209074	-0.423573752	Yes
430	1.246772172	-0.050379165	No
431	-1.110710939	-0.421519617	Yes
432	0.939714472	-0.564606007	No
433	-1.069651147	-0.061753168	Yes
435	0.266754247	-0.380030137	Yes
436	0.923351156	-0.535106926	No
437	0.192806712	-0.850262646	Yes
438	0.299328429	0.919881094	Yes
439	-0.097482861	-0.696675954	Yes
440	-0.474624944	-0.58514278	Yes
441	-0.474624944	-0.58514278	Yes
442	-0.758293398	-0.868390264	Yes
443	-0.089876921	-0.259950589	Yes
444	-0.092755253	-0.482383573	Yes
445	-0.454114029	-0.576596407	Yes
446	0.566719988	-0.415027378	No
447	1.561786287	0.131371616	No
448	-1.426282315	0.05832638	Yes
449	-0.101696489	-1.023467053	Yes
450	-0.804210499	0.754304002	Yes
451	-0.795803598	1.057280982	Yes
452	0.205361212	-0.509240212	Yes
453	-0.478838571	-0.91193388	Yes
454	-0.803653238	0.092707224	Yes
455	1.561786287	0.131371616	No
456	-0.804210499	0.754304002	Yes
457	0.281980292	0.075820634	Yes
458	0.877006495	-0.447568979	No
459	0.877006495	-0.447568979	No
461	1.184644205	0.24290479	No
462	0.939714472	-0.564606007	No
463	-1.146827488	0.014782764	Yes
464	-0.086754889	0.490330787	Yes
466	-0.434444822	1.151493815	Yes
467	1.246772172	-0.050379165	No
469	-0.092755253	-0.482383573	Yes
471	-1.069651147	-0.061753168	Yes
472	1.238365271	-0.353356145	No
473	0.56143512	0.032277018	No
474	0.570376355	0.573360499	No
476	0.893797371	1.05808826	No
477	-1.119224614	0.572553221	Yes
479	0.569948795	-0.96179582	Yes
480	1.561786287	0.131371616	No
482	-1.426282315	0.05832638	Yes

483	-0.451235697	-0.354163423	Yes
484	-0.094604529	-0.47424297	Yes
485	-1.146827488	0.014782764	Yes
486	-0.754079771	-0.541599165	Yes
487	-0.831813372	0.196533545	Yes
489	-0.451235697	-0.354163423	Yes
490	1.200427511	0.037158782	No
492	-1.426282315	0.05832638	Yes
493	-0.162839635	0.143376442	Yes
494	1.246772172	-0.050379165	No
495	-0.787847184	0.724804921	Yes
496	-1.426282315	0.05832638	Yes
497	0.532995856	0.302278648	No
498	-1.064923539	0.152539213	Yes
499	0.167957787	0.119381216	Yes
500	-0.451235697	-0.354163423	Yes
501	-1.426282315	0.05832638	Yes
502	0.574676403	-0.747503439	Yes
503	-0.823406472	0.499510525	Yes
504	-0.795696824	-0.239768836	Yes
505	0.566162728	0.2465694	No
507	0.163810188	0.081335762	Yes
508	0.163810188	0.081335762	Yes
509	1.205155119	0.251451163	No
511	-0.127814681	0.130564338	Yes
512	-0.790196319	-0.105296783	Yes
513	1.238365271	-0.353356145	No
514	0.205361212	-0.509240212	Yes
515	-1.426282315	0.05832638	Yes
516	0.922793896	0.126489852	No
517	0.922793896	0.126489852	No
519	-1.426282315	0.05832638	Yes
520	-0.696722692	-0.500077441	Yes
521	0.616293456	-1.049333768	Yes
522	-0.119430707	1.333244596	Yes
523	-0.708292371	0.032459666	Yes
524	-0.839769787	0.529009606	Yes
525	-1.426282315	0.05832638	Yes
526	-0.165218107	0.759185766	Yes
528	-1.073864774	-0.388544267	Yes
529	-0.696722692	-0.500077441	Yes
531	1.561786287	0.131371616	No
532	-1.064923539	0.152539213	Yes
533	0.920972684	0.080702398	No
536	1.28233146	0.174915231	No
537	1.238365271	-0.353356145	No
539	-0.454114029	-0.576596407	Yes

540	1.200427511	0.037158782	No
542	0.259662287	-0.92925422	Yes
544	-1.426282315	0.05832638	Yes
545	-0.376937688	-0.653132339	Yes
546	1.561786287	0.131371616	No
547	-0.480232222	0.577434985	Yes
548	0.151640146	0.087020723	Yes
549	-1.426282315	0.05832638	Yes
550	-0.105416527	0.473643811	Yes
551	0.290493968	-0.918252205	Yes
552	0.884856136	0.517004779	No
553	-0.17178087	-0.397707038	Yes
555	0.230665763	0.002344188	Yes
556	-0.804210499	0.754304002	Yes
557	-0.113266168	-0.490929947	Yes
558	-0.754079771	-0.541599165	Yes
559	0.885413396	-0.144591999	No
560	-1.426282315	0.05832638	Yes
563	-0.200777395	0.53389137	Yes
564	1.28233146	0.174915231	No
567	-0.437778779	-0.55216743	Yes
568	0.295221575	-0.703959824	Yes
569	-1.426282315	0.05832638	Yes
570	-0.754079771	-0.541599165	Yes
572	0.923351156	-0.535106926	No
573	0.97527376	-0.33931161	No
574	0.252983768	1.007419042	Yes
575	-0.36436044	0.5257338	Yes
579	-0.172338131	0.26388974	Yes
580	-0.790196319	-0.105296783	Yes
581	-0.159717603	0.893657819	Yes
582	-1.426282315	0.05832638	Yes
583	0.894354632	0.396491482	No
584	1.561786287	0.131371616	No
585	-0.795803598	1.057280982	Yes
586	0.205361212	-0.509240212	Yes
587	-0.17178087	-0.397707038	Yes
588	-0.795803598	1.057280982	Yes
590	-0.101696489	-1.023467053	Yes
591	-0.524198411	0.049163609	Yes
592	0.192806712	-0.850262646	Yes
593	0.220409586	-0.292492189	Yes
594	1.246772172	-0.050379165	No
596	1.28233146	0.174915231	No
598	-0.831256112	-0.465063233	Yes
599	-1.146827488	0.014782764	Yes
600	-0.110210164	-0.029394215	Yes

601	-1.426282315	0.05832638	Yes
602	0.610686177	0.113243998	No
603	0.614899804	0.440035097	No
604	-1.426282315	0.05832638	Yes
605	-1.426282315	0.05832638	Yes
606	-1.426282315	0.05832638	Yes
607	0.517212549	0.508024656	No
608	0.525168964	0.175548595	Yes
609	0.525168964	0.175548595	Yes
610	-1.426282315	0.05832638	Yes
611	-0.434872382	-0.383662503	Yes
612	0.660259644	-0.521062391	Yes
614	-0.795696824	-0.239768836	Yes
615	-0.05730274	1.039960642	Yes
617	-1.110710939	-0.421519617	Yes
618	-0.803653238	0.092707224	Yes
619	-1.110710939	-0.421519617	Yes
620	-1.110710939	-0.421519617	Yes
621	-1.426282315	0.05832638	Yes
622	-0.790196319	-0.105296783	Yes
623	-1.426282315	0.05832638	Yes
624	-1.426282315	0.05832638	Yes
625	-0.478838571	-0.91193388	Yes
626	-1.426282315	0.05832638	Yes
627	0.923351156	-0.535106926	No
628	0.56199238	-0.62931976	Yes
629	0.967317345	-0.00683555	No
630	0.614899804	0.440035097	Yes
631	-1.426282315	0.05832638	Yes
633	0.151640146	0.087020723	Yes
634	0.529316563	0.213594049	Yes
636	-0.487352246	0.082138959	Yes
637	1.238365271	-0.353356145	No
638	0.920972684	0.080702398	No
639	0.925700292	0.294994779	No
640	-0.43917243	0.937201434	Yes
641	0.54855821	0.406527953	Yes
642	1.561786287	0.131371616	No
644	0.532995856	0.302278648	No
646	-0.201334656	1.195488148	Yes
648	-1.426282315	0.05832638	Yes
650	0.614342544	1.101631875	Yes
651	0.532438595	0.963875426	Yes
652	-0.478411011	0.623222439	Yes
653	1.561786287	0.131371616	No
654	-0.462627704	0.417476432	Yes
655	-0.139206688	0.902204193	Yes

656	-0.074093615	-0.465696597	Yes
657	-1.146827488	0.014782764	Yes
658	-1.426282315	0.05832638	Yes
659	-0.43917243	0.937201434	Yes
660	-0.516348771	1.013737367	Yes
661	-1.146827488	0.014782764	Yes
662	1.561786287	0.131371616	No
663	-0.147056329	-0.062369565	Yes
665	1.200427511	0.037158782	No
666	-0.758293398	-0.868390264	Yes
667	1.561786287	0.131371616	No
668	-0.687781456	0.041006039	Yes
669	-1.146827488	0.014782764	Yes
670	-0.831256112	-0.465063233	Yes
671	0.616293456	-1.049333768	Yes
672	-0.162839635	0.143376442	Yes
674	-1.119224614	0.572553221	Yes
675	-0.766807073	0.125682574	Yes
676	1.28233146	0.174915231	No
677	-0.376937688	-0.653132339	Yes
679	-0.516348771	1.013737367	Yes
680	-0.070437248	0.522691281	Yes
681	-1.064923539	0.152539213	Yes
682	-1.426282315	0.05832638	Yes
684	-0.451235697	-0.354163423	Yes
685	-1.110710939	-0.421519617	Yes
686	-0.757865838	0.666766055	Yes
688	1.246772172	-0.050379165	No
689	-0.028306215	0.108362234	Yes
690	-1.426282315	0.05832638	Yes
691	-0.478838571	-0.91193388	Yes
694	-1.049140232	-0.053206794	Yes
696	1.254728587	-0.382855226	No
697	0.898097419	-0.262775678	No
698	-0.069879988	-0.138905497	Yes
700	-0.40166223	-0.988469812	Yes
702	1.184644205	0.24290479	No
703	0.939157211	0.096990771	No
704	-0.839769787	0.529009606	Yes
705	1.561786287	0.131371616	No
706	0.920972684	0.080702398	No
707	0.193791533	0.023296895	Yes
708	-0.754079771	-0.541599165	Yes
710	-0.434444822	1.151493815	Yes
711	0.570933616	-0.088236279	No
712	-0.785468712	0.108995598	Yes
713	-1.426282315	0.05832638	Yes

715	1.200427511	0.037158782	No
716	1.561786287	0.131371616	No
717	0.225938155	-0.211948193	Yes
718	-0.831256112	-0.465063233	Yes
719	0.931307571	-0.867582987	No
720	0.57889003	-0.42071234	No
721	0.931307571	-0.867582987	No
722	-0.443279283	-0.686639483	Yes
726	1.28233146	0.174915231	No
727	1.28233146	0.174915231	No
728	-1.426282315	0.05832638	Yes
729	-1.110710939	-0.421519617	Yes
730	-0.480232222	0.577434985	Yes
731	0.828013037	0.362984337	No
732	-0.516348771	1.013737367	Yes
734	-0.749352163	-0.327306784	Yes
737	0.143233245	-0.215956257	Yes
738	1.238365271	-0.353356145	No
739	-1.426282315	0.05832638	Yes
740	1.184644205	0.24290479	No
741	-0.417267864	-0.543621056	Yes
743	-0.066243975	0.498877161	Yes
744	-0.725719216	0.431520967	Yes
745	-1.146827488	0.014782764	Yes
746	-0.413054237	-0.216829957	Yes
747	1.561786287	0.131371616	No
748	-0.839769787	0.529009606	Yes
749	-1.110710939	-0.421519617	Yes
750	-0.17178087	-0.397707038	Yes
751	0.151640146	0.087020723	Yes
752	-1.119224614	0.572553221	Yes
753	-0.758293398	-0.868390264	Yes
754	1.561786287	0.131371616	No
755	-0.734126117	0.128543987	Yes
756	-1.426282315	0.05832638	Yes
757	0.643896329	-0.491563311	No
760	-0.687781456	0.041006039	Yes
761	0.894354632	0.396491482	No
762	-0.396934622	-0.774177431	Yes
763	-1.426282315	0.05832638	Yes
764	-0.066243975	0.498877161	Yes
766	-0.048817129	0.09981586	Yes
767	-1.110710939	-0.421519617	Yes
768	-0.795803598	1.057280982	Yes
769	1.246772172	-0.050379165	No
771	1.561786287	0.131371616	No
772	-1.426282315	0.05832638	Yes

773	-1.110710939	-0.421519617	Yes
774	0.56199238	-0.62931976	Yes
776	-0.746230131	0.422974593	Yes
777	0.660259644	-0.521062391	Yes
778	-1.119224614	0.572553221	Yes
779	-0.066243975	0.498877161	Yes
783	-1.426282315	0.05832638	Yes
784	0.967317345	-0.00683555	No
785	1.246772172	-0.050379165	No
786	-0.795803598	1.057280982	Yes
787	1.238365271	-0.353356145	No
788	-0.118873446	0.671647818	Yes
789	-0.055909088	-0.449408223	Yes
792	-0.831256112	-0.465063233	Yes
793	0.56199238	-0.62931976	No
794	-1.146827488	0.014782764	Yes
795	-0.790196319	-0.105296783	Yes
796	-0.086754889	0.490330787	Yes
797	-1.426282315	0.05832638	Yes
798	0.923351156	-0.535106926	No
800	-1.110710939	-0.421519617	Yes
801	-0.454114029	-0.576596407	Yes
802	0.20957484	-0.182449113	Yes
803	-0.451235697	-0.354163423	Yes
804	-1.119224614	0.572553221	Yes
805	1.561786287	0.131371616	No
806	-0.478838571	-0.91193388	Yes
807	-0.478838571	-0.91193388	Yes
808	-0.478838571	-0.91193388	Yes
810	-0.790196319	-0.105296783	Yes
811	1.246772172	-0.050379165	No
813	-0.742082532	0.461020047	Yes
814	-1.426282315	0.05832638	Yes
817	-0.089876921	-0.259950589	Yes
818	0.569948795	-0.96179582	No
820	-0.451235697	-0.354163423	Yes
821	-0.123601054	0.457355437	Yes
822	0.516655288	1.169621434	No
823	0.508271314	-0.033058825	No
824	0.239151373	-0.937800594	Yes
825	1.28233146	0.174915231	No
826	1.561786287	0.131371616	No
827	0.923351156	-0.535106926	No
828	1.246772172	-0.050379165	No
829	-1.426282315	0.05832638	Yes
831	0.257711376	1.221711423	Yes
832	-1.426282315	0.05832638	Yes

834	1.209368747	0.578242263	No
835	1.209368747	0.578242263	No
836	1.209368747	0.578242263	No
837	-1.426282315	0.05832638	Yes
838	-0.451235697	-0.354163423	Yes
839	1.561786287	0.131371616	No
840	-0.105996536	0.297396884	Yes
841	0.336838628	-1.005790152	Yes
842	0.898097419	-0.262775678	No
843	-1.426282315	0.05832638	Yes
844	0.15959656	-0.245455338	Yes
845	-0.795803598	1.057280982	Yes
846	1.246772172	-0.050379165	No
848	0.56199238	-0.62931976	Yes
850	0.228816487	0.01048479	Yes
852	-0.433051171	-0.337875049	Yes
853	-0.749352163	-0.327306784	Yes
854	0.211923975	0.647652592	Yes
855	0.893369811	-0.477068059	No
857	-0.101696489	-1.023467053	Yes
858	-0.516348771	1.013737367	Yes
859	-1.426282315	0.05832638	Yes
860	-1.426282315	0.05832638	Yes
861	-1.426282315	0.05832638	Yes
862	-1.110710939	-0.421519617	Yes
863	-1.1112682	0.240077161	Yes
864	-0.16393123	0.56686672	Yes
865	0.881734103	-0.233276597	No
866	-0.831256112	-0.465063233	Yes
867	-1.426282315	0.05832638	Yes
868	1.561786287	0.131371616	No
869	-0.331150288	-0.079073508	Yes
870	-0.430658755	-0.056871404	Yes
872	-1.426282315	0.05832638	Yes
873	-0.40166223	-0.988469812	Yes
874	1.561786287	0.131371616	No
875	0.237200461	1.213165049	Yes
876	-0.516348771	1.013737367	Yes
877	-1.426282315	0.05832638	Yes
878	0.11423672	0.715642151	Yes
879	-0.758293398	-0.868390264	Yes
880	1.238365271	-0.353356145	No
882	0.616293456	-1.049333768	No
883	-0.785468712	0.108995598	Yes
884	-0.10309014	0.465901811	Yes
885	1.561786287	0.131371616	No
886	-1.426282315	0.05832638	Yes

887	0.257711376	1.221711423	Yes
888	-0.109630155	0.146852712	Yes
889	0.582526043	0.217070319	No
890	0.582526043	0.217070319	No
891	-1.426282315	0.05832638	Yes
892	1.561786287	0.131371616	No
893	0.211923975	0.647652592	Yes
894	-1.426282315	0.05832638	Yes
895	0.249327401	0.019031164	Yes
896	1.561786287	0.131371616	No
897	-0.746230131	0.422974593	Yes
898	-1.426282315	0.05832638	Yes
899	-1.069651147	-0.061753168	Yes
901	-0.762593446	0.452473674	Yes
902	-0.154989995	1.1079502	Yes
903	-0.790196319	-0.105296783	Yes
905	-0.396934622	-0.774177431	Yes
908	-1.426282315	0.05832638	Yes
909	0.239151373	-0.937800594	Yes
910	-1.426282315	0.05832638	Yes
911	-0.076420003	-0.457954597	Yes
913	0.25493468	-1.143546601	Yes
914	1.184644205	0.24290479	No
916	-0.839769787	0.529009606	Yes
917	1.246772172	-0.050379165	No
918	-1.119224614	0.572553221	Yes
919	-1.102861299	0.54305414	Yes
920	0.890141004	0.069700382	No
921	1.28233146	0.174915231	No
922	-0.331150288	-0.079073508	Yes
924	-0.44702207	-0.027372323	Yes
926	-0.478838571	-0.91193388	Yes
927	0.193234272	0.684893673	Yes
928	0.537166203	1.178167807	No
929	0.508805648	0.205047676	No
930	0.16002412	1.289700981	Yes
931	0.537166203	1.178167807	No
935	0.92991392	0.621785878	No
936	-0.831256112	-0.465063233	Yes
937	0.564341516	0.200781945	Yes
938	1.246214912	0.611217613	No
939	0.607779781	-0.055260929	Yes
940	0.922793896	0.126489852	No
941	1.238365271	-0.353356145	No
943	1.561786287	0.131371616	No
944	-1.426282315	0.05832638	Yes
945	0.569948795	-0.96179582	Yes

947	-0.396934622	-0.774177431	Yes
949	1.246772172	-0.050379165	No
950	0.890141004	0.069700382	No
951	-1.049140232	-0.053206794	Yes
952	-1.049140232	-0.053206794	Yes
954	0.2409205	-0.283945816	Yes
955	0.881734103	-0.233276597	No
956	-1.426282315	0.05832638	Yes
957	-0.016136173	0.102677273	Yes
958	-1.110710939	-0.421519617	Yes
959	0.939714472	-0.564606007	No
960	0.221724527	-0.538739293	Yes
961	-0.454114029	-0.576596407	Yes
962	-0.434444822	1.151493815	Yes
963	-0.754079771	-0.541599165	Yes
965	-0.839769787	0.529009606	Yes
966	-0.385451363	0.3409405	Yes
967	-0.804210499	0.754304002	Yes
968	0.546209074	-0.423573752	No
969	0.564341516	0.200781945	Yes
970	0.923351156	-0.535106926	No
971	0.643896329	-0.491563311	Yes
973	1.209368747	0.578242263	No
974	-0.418554741	-0.35130201	Yes
975	0.266754247	-0.380030137	Yes
976	0.021267253	-0.525944155	Yes
977	0.57889003	-0.42071234	Yes
978	0.885413396	-0.144591999	No
980	-0.769685405	-0.09675041	Yes
981	-0.487352246	0.082138959	Yes
982	0.258268636	0.560114645	Yes
983	-1.146827488	0.014782764	Yes
984	-1.426282315	0.05832638	Yes
986	-0.479395832	-0.250337102	Yes
987	0.885947731	0.093514502	No
988	-1.146827488	0.014782764	Yes
989	0.610686177	0.113243998	Yes
990	-1.119224614	0.572553221	Yes
991	-0.839769787	0.529009606	Yes
992	-0.769685405	-0.09675041	Yes
993	0.570933616	-0.088236279	Yes
994	-1.426282315	0.05832638	Yes
996	1.561786287	0.131371616	No
997	1.254728587	-0.382855226	No
999	-0.478411011	0.623222439	Yes
1000	0.225380894	0.449648585	Yes
1001	1.200427511	0.037158782	No

1002	0.2409205	-0.283945816	Yes
1003	0.249327401	0.019031164	Yes
1004	-0.162839635	0.143376442	Yes
1005	-1.049140232	-0.053206794	Yes
1006	-0.101268928	0.511689265	Yes
1007	-0.101268928	0.511689265	Yes
1009	-1.426282315	0.05832638	Yes
1013	1.246772172	-0.050379165	No
1014	-1.426282315	0.05832638	Yes
1017	0.303628476	-0.400982844	Yes
1018	-0.024520148	-1.100002986	Yes
1019	0.516655288	1.169621434	No
1020	-0.101696489	-1.023467053	Yes
1021	0.828013037	0.362984337	No
1022	-0.725719216	0.431520967	Yes
1023	1.561786287	0.131371616	No
1024	0.651852744	-0.824039371	Yes
1025	1.561786287	0.131371616	No
1026	0.967317345	-0.00683555	No
1027	1.561786287	0.131371616	No
1028	-0.119430707	1.333244596	Yes
1029	-0.201334656	1.195488148	Yes
1030	-0.754079771	-0.541599165	Yes
1031	0.925700292	0.294994779	No
1032	-0.024520148	-1.100002986	Yes
1033	-0.101268928	0.511689265	Yes
1034	1.200427511	0.037158782	No
1036	-0.069879988	-0.138905497	Yes
1037	-0.376937688	-0.653132339	Yes
1038	1.246214912	0.611217613	No
1039	0.239151373	-0.937800594	Yes
1040	0.151640146	0.087020723	Yes
1041	1.561786287	0.131371616	No
1042	0.894354632	0.396491482	No
1043	-0.708292371	0.032459666	Yes
1044	-1.426282315	0.05832638	Yes
1045	-1.426282315	0.05832638	Yes
1046	-1.426282315	0.05832638	Yes
1048	-0.101696489	-1.023467053	Yes
1050	1.561786287	0.131371616	No
1051	-0.154989995	1.1079502	Yes
1052	-1.119224614	0.572553221	Yes
1053	0.958910444	-0.30981253	No
1054	-0.443279283	-0.686639483	Yes
